# Surface Topography Improvement of Micro-Milled Inconel 718 Slots Through Abrasive Deburring: Comparison Between Polishing and Sanding

**DOI:** 10.3390/mi17080951

**Published:** 2026-08-11

**Authors:** Gabriel de Paiva Silva, Marcelo Lopes Araujo, Raphael Lima de Paiva, Maksym Ziberov, Déborah de Oliveira

**Affiliations:** 1 École Nationale Supérieure d’Arts et Métiers, LAMPA, 2 Bd du Ronceray, 49035 Angers, France; dpaivagabriel@gmail.com; 2 Department of Mechanical Engineering, University of Brasilia, Asa Norte, Brasília 70910-900, DF, Brazil; marcelo.lps.araujo@gmail.com (M.L.A.); mziberov@unb.br (M.Z.);; 3 School of Mechanical Engineering, Federal University of Piauí, Ministro Petrônio Portella Campus, Ininga, Teresina 64049-550, PI, Brazil

**Keywords:** micro milling, abrasive deburring, burr formation, surface topography, Inconel 718

## Abstract

Burr formation remains a major challenge in micro milling due to reduced tool dimensions and size-effect phenomena, particularly when machining difficult-to-cut materials such as Inconel 718. This study compares the effectiveness of two abrasive deburring processes, polishing and sanding, applied to micro-milled slots, focusing on burr removal and surface topography modification. Twelve 15 mm long slots were machined using a 400 µm diameter TiAlN-coated micro end mill under constant cutting conditions. Deburring was performed for 1, 2, and 4 min for polishing and for 30 s for sanding. Surface topography and burr height were evaluated before and after each process. The results revealed significant burr formation, with burr heights reaching the same order of magnitude as the axial depth of cut and higher values on the down-milling side. Polishing showed limited deburring capability, primarily promoting burr deformation while reducing surface roughness by up to 21%. In contrast, sanding reduced total height of the burrs by up to 99% and generated flatter edge morphologies, although surface roughness increased by up to 19%. These findings highlight the trade-off between burr control and surface quality in the abrasive post-processing of micro-milled Inconel 718 features.

## 1. Introduction

Structures and features in small-dimension mechanical components can be fabricated using reduced cutting tools conventionally used in machining operations [1,2]. However, achieving high levels of surface quality, along with high process efficiency, remains a major challenge in micro milling. The accuracy of processing and the surface quality of precision components are challenging factors that hinder their further advancement [3,4]. According to Mativenga [5], micro milling can be defined as the use of mechanical micro tools to manufacture devices or features whose dimensions are in the micrometer range (1–999 µm). Câmara et al. [6] define that in micro-milling processes, the diameter of the cutting tool should be less than 1000 µm. Micro milling is often used for fabricating microscopic cavities, slots, and tridimensional features in metals and other materials. Industries that employ this process include biomedical, microelectronics, aerospace and nanotechnology [7,8].

The chip formation mechanism has important particularities in micro milling, and the physical principles used for analyzing metal cutting in traditional (macro-scale) machining are not applicable. This is mainly attributed to the size effect, which is caused by the similar size of the tool edge radius and the minimum uncut chip thickness. In micro milling, the order of magnitude of quantities such as cutting forces, feed rate and depth of cut are considerably smaller than in conventional machining [5]. The size of the radius of the micro mill is not negligible and, therefore, the tool cannot be treated as perfectly sharp [9]. As a consequence, during micro cutting, a large amount of workpiece material suffers only plastic deformation along the round tool instead of being properly sheared and removed in the form of chips [10]. This often results in poor surface quality, with problems such as plowing marks, tolerance errors, surface roughness deterioration and large burrs.

Size effect directly impacts the fundamental mechanisms of the cutting process in micro milling, influencing the quality and characteristics of the manufactured part [11]. The performance of micro mills is significantly affected by minor vibrations and excessive forces, which can reduce tool life and compromise the precision of miniaturized component tolerances [12,13]. The plowing originating from size effect causes the deformed workpiece material to accumulate on the borders of the micro-milled area, resulting in burrs that can get four times as high as the minimum chip thickness [14]. In micro-milled slots, the height of top burrs can exceed the slot depth by up to 156%, as observed by Silva and da Silva [15] in micro milling of UNS S32205 duplex stainless steel. As a result, these burrs can adversely affect dimensional accuracy, parts assembly, component reliability, cost, safety and appearance [16,17]. Thus, burr formation can be considered a major problem in micro milling, and the removal/control of the burrs represents an important research topic for improving process performance and quality of micro machined components [18,19,20].

There are several techniques for deburring micro-machined pieces. However, removing burrs from micro components is especially challenging, as conventional deburring methods used in traditional macroscale milling often lack the required precision at the microscale level [21]. Sun et al. [22] conducted micro-milling experiments on copper using a 0.5 mm diameter micro-end mill and evaluated burr formation under various cutting conditions. Their results indicate that, with constant spindle speed and feed rate, burr size increased with depth of cut. Likewise, maintaining constant spindle speed and depth of cut, higher feed rates resulted in greater burr formation. Gillespie [17] comments that the most common deburring techniques are typically the simplest and most cost-effective, such as abrasive or manual methods, which can be time-consuming and require training for developing the necessary skills [23]. It is possible to decrease burr formation with single-factor analyses and multi-factor optimization of parameters such as axial depth of cut, feed per tooth and spindle speed, as well as with further post-processing procedures [24]. In addition, several techniques for removing burrs or suppressing burr formation have been analyzed by different researchers.

Matsumura et al. [25] investigated different methods to remove burrs from micro pillars fabricated on an aluminum plate. The authors concluded that a combination of polishing and water jet finishing was necessary to completely deburr the pillars since each individual method could not remove burrs in all directions. Kienzler et al. [26] analyzed two alternative deburring methods: abrasive peening and ultrasonic wet peening. Both of these processes involve propelling a peening medium (typically microscopic beads) at high speeds toward the workpiece surface, removing burrs from the target area. The authors used these methods to deburr micro slots on 30CrMo6 steel samples. Main results indicated that, although these techniques can reduce burr size in micro components, the workpiece surface topography may also be adversely affected, since many burrs are only displaced instead of fully removed.

Kumar et al. [27] proposed an ultrasonic-assisted abrasive micro-deburring process for removing burrs from high-aspect-ratio micropillars made of copper and aluminum 6061. Successful deburring of the micro-milled pillars with a 5:1 aspect ratio was achieved by the authors, without compromising the structural integrity of the features. The primary mechanism of burr removal was found to be the impact of abrasive particles, with minimal contribution from liquid cavitation. Thus, the authors suggest that pure water cavitation plays a minor role in deburring, but it is not as effective as the addition of abrasive particles, resulting in a significantly shorter processing time as compared to the literature. Jeong et al. [28] proposed the use of micro electrical discharge machining (micro EDM) to remove burrs from 200 µm wide micro walls manufactured from three workpiece materials: the AL6061 aluminum alloy, pure copper, and the AISI 304 stainless steel. The authors comment that micro EDM uses thermal energy without mechanical contact between the tool and workpiece, being an effective method for deburring conductive materials regardless of their hardness. It was found that burrs with dimensions up to 1000 µm of height were successfully removed with an error of 1 µm.

The nickel superalloy Inconel 718 (In718) is known for its excellent mechanical strength, as well as its resistance to wear, corrosion, and creep at elevated temperatures. Because of these properties, it is widely used in the aerospace and nuclear sectors [29]. However, this alloy is also notorious for being difficult to machine. Micro-milling In718 presents significant challenges, including abrasive flank wear and localized chipping of the tool cutting edge [30]. Additionally, due to the ductility of In718 and its strong work-hardening tendency, burr formation represents a significant challenge, with large burrs commonly observed after micro milling. If this issue is not effectively controlled or mitigated, it may compromise quality control and component performance. There are few research studies covering deburring in micro milling of In718. For instance, Fang et al. [31] showed that ultrasonic vibration-assisted micro milling was able to suppress burr size, decrease cutting forces and, consequently, improve surface quality of Inconel 718 micro grooves.

Silva et al. [32] demonstrated that abrasive sanding can remove burrs from micro-milled Inconel 718 slots with high efficiency. However, the study focused primarily on burr height reduction and did not investigate how different abrasive finishing mechanisms influence surface integrity, burr morphology, or the statistical significance of the process variables. In particular, no comparison was performed between fixed-abrasive sanding and free-abrasive polishing, which exhibit distinct material removal mechanisms and may lead to different balances between burr removal efficiency and surface quality.

In this context, the present work aims to compare the efficiency of different abrasive deburring techniques—sanding and polishing—in removing burrs from micro-milled slots in In718 samples. Thus, unlike the previous study, which focused exclusively on the burr removal capability of abrasive sanding, the present work establishes a comparative framework between two-body and three-body abrasive deburring techniques by combining quantitative burr height measurements, burr morphology characterization, surface integrity evaluation, and statistical assessment.

## 2. Materials and Methods

### 2.1. Micro-Milling Trials: Workpiece Material, Machine Tool, Cutting Parameters, and Cutting Tool

To study the deburring process, it was necessary to produce micro slots. For this, the material selected was the nickel superalloy In718, which combines a tensile strength of 1275 MPa, yield strength of 1034 MPa, Young’s modulus of 200 GPa, hardness of 40 HRc and a thermal conductivity of 11.4 W/mk [33]. Those are some of the properties that make it desirable for a wide range of applications. Added to this, its capacity to operate at high temperatures and high chemical resistance also enables some critical applications [34]. This material has an austenitic microstructure with hard precipitates and carbides, which influences the micro cut [35]. The ductility of the matrix makes it susceptible to high-dimensional burrs [12], thus being an important material for analyzing deburring. The widely known low machinability of this alloy can further interfere with both burr formation and its removal.

The machine used for manufacturing the micro slots was the precision CNC Mini-mill/GX (Minitech Machinery Corporation, Norcross, GA, USA), with 0.10 µm position resolution. The cutting parameters were a spindle speed (n) of 20,000 rpm, an axial depth of cut (a_p_) of 40 µm, a radial depth of cut (a_e_) of 400 µm, equal to the tool diameter, and a feed per tooth of 5 µm/tooth. The cutting tool was a 400 µm TiAlN-coated carbide micro mill, with two teeth from Mitsubishi (MS2MSD0040) (Tokyo, Japan). As the focus of the work is deburring, the cutting parameters were not varied. For all machining experiments, the cutting fluid Coolube 2210EP (UNIST, Inc., Grand Rapids, MI, USA)was applied at a 270 mL/h flow rate and 0.25 MPa. Four In718 samples were used in this study, and twelve micro slots of 15 mm in length were machined on each sample. A new cutting tool was employed for each sample, as tool wear greatly influences burr formation [30]; therefore, each tool machined twelve micro slots.

### 2.2. Deburring Procedures

**Figure 1:**
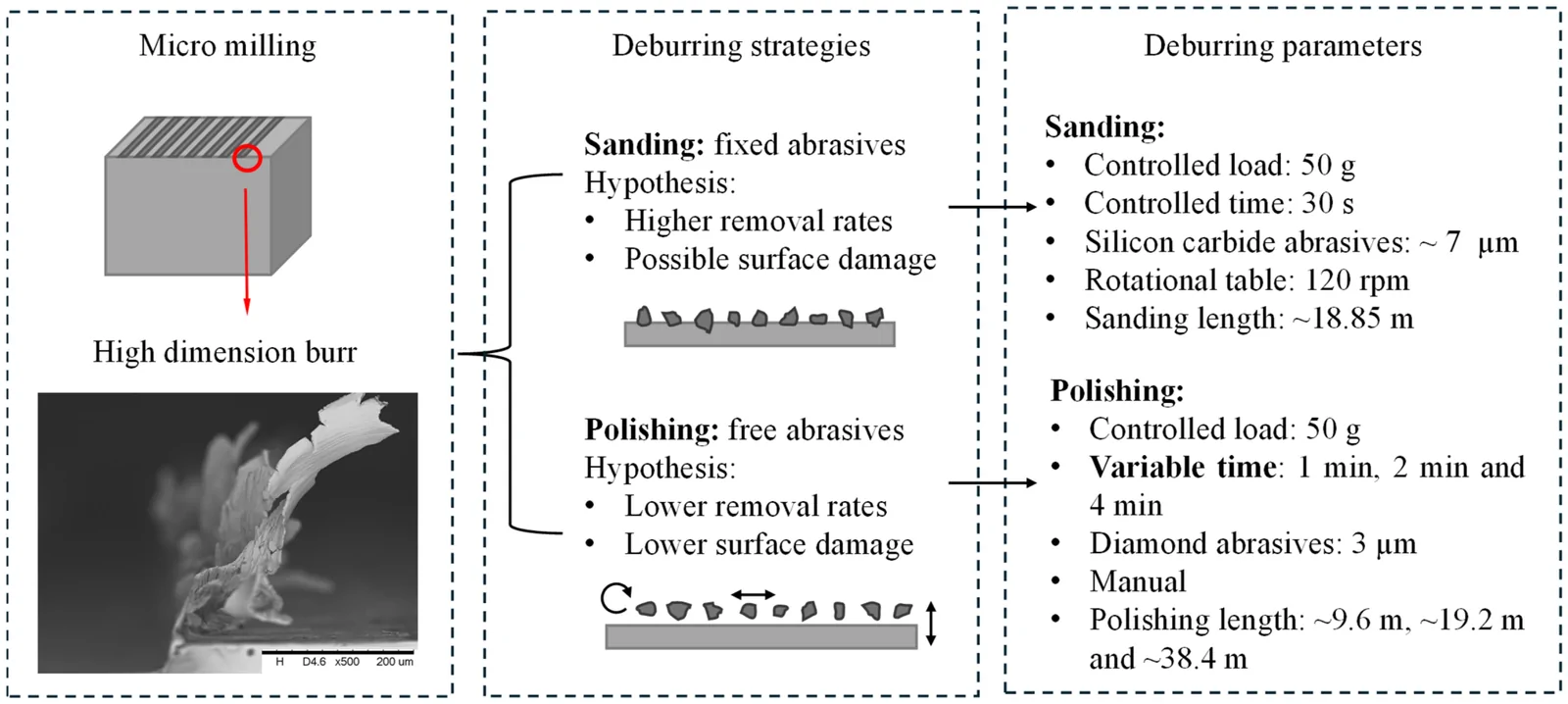
Scheme of deburring strategies and parameters.

The samples were divided into two groups: two were subjected to the polishing deburring process, while the other two underwent the sanding deburring process. Accordingly, two samples (trial and replica) were assigned to each method to increase reliability. The parameters and specific conditions of both deburring techniques are presented in Figure 1. Sanding was performed using a conventional metallographic grinder operating at 120 rpm, based on the work of Silva et al. [32], where the deburring process was detailed. In their work, the authors noticed that, besides being effective for burr removal, the fixed abrasives damaged the surface during the sanding process. Thus, polishing was selected to compare with this other abrasive process. Since polishing is performed with a smooth polishing cloth, it does not usually interfere with the surface geometry. Nevertheless, it is important to analyze processes with low removal rates such as polishing because they could be insufficient for higher resistance materials such as In718.

Although polishing was performed manually, two key process parameters were controlled to improve repeatability: the applied normal load and the total polishing time. The applied normal load of 50 g was controlled using a calibrated weight placed on top of the specimen, maintaining the same load used in the sanding experiments. The sliding distance of the samples during polishing was quantified, corresponding to approximately 4.8 m every 30 s. Therefore, the total polishing trajectories were approximately 9.6, 19.2 and 38.4 m for the 1, 2 and 4 min conditions, respectively. For comparison, the sanding process, performed on a rotary table operating at 120 rpm with a 5 cm radius, produced a total sliding distance of approximately 18.85 m during 30 s of processing [32]. Consequently, the 2 min polishing condition (19.2 m) provided a sliding distance comparable to that of the sanding process, allowing the comparison to focus primarily on the different abrasive mechanisms (three-body abrasion in polishing and fixed-abrasive two-body abrasion in sanding), rather than on the total interaction length. It is also worth noting that manual polishing remains a common practice in industrial finishing operations, particularly for complex geometries and localized surface correction. Moreover, all polishing tests were performed by the same operator following the same polishing procedure and under controlled polishing distance, processing time and normal load conditions, in order to minimize operator-induced variability.

Nevertheless, the manual execution may introduce operator-dependent variations, such as stroke uniformity and velocity fluctuations, which were not completely eliminated in the present work and should be considered a limitation of the study. Future investigations employing automated polishing systems could further reduce operator influence and improve process repeatability.

### 2.3. Burr and Surface Roughness Measurements

**Figure 2:**
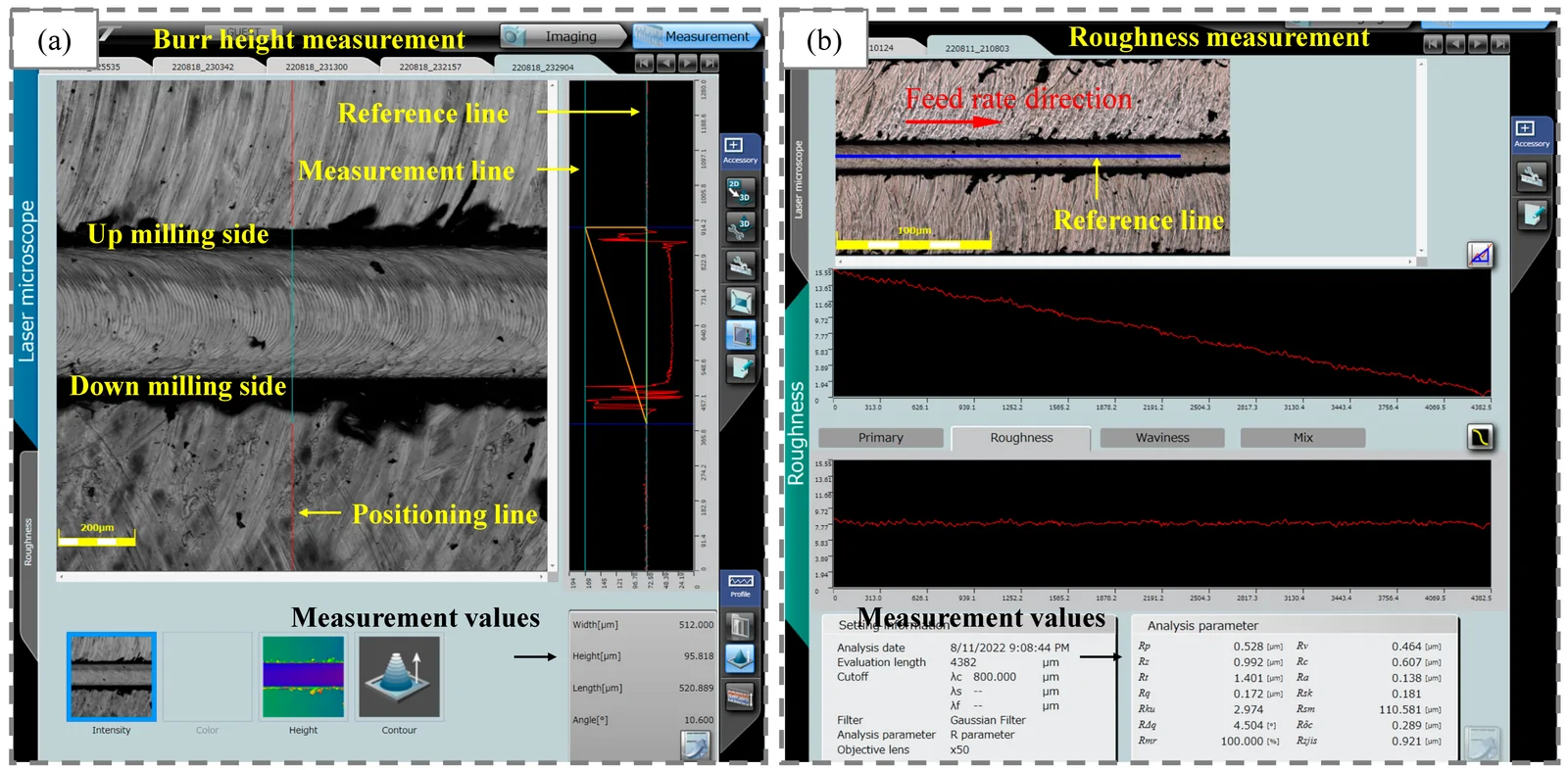
Measurement methodology representation for burr height (**a**) and surface roughness (**b**).

The main output variable was the burr height, which was obtained for both up-milling and down-milling sides. The burrs heights were measured with the aid of an Olympus Lext OLS4100 Confocal Laser Scanning Microscope (Tokio, Japan). In Figure 2, one can observe the microscope measurement interface after scanning the topography of the part with the laser. In the microscope interface, it was possible to select the point of the positioning line. For this section, a reference line was positioned on the part surface and a measurement line positioned on the top of the burr on the side that was being measured. After positioning, the distance was evaluated and registered by the microscope. For each slot and each burr side, five measurements were performed.

Similarly, the surface roughness was also obtained by the OLS4100; the measurements were made in the same direction as the feed rate, therefore aligned with the 15 mm length of the machined slot (reference line Figure 2b). A Gaussian filter and a cut-off of 0.8 mm were employed in accordance with ISO 4288 [36]. Three measurements were made for each slot, and parameter Ra, as defined by ISO 4287 [37], was considered for analysis.

A reduced nomenclature was adopted for better compression. Thus, “Up-Milling Burr Height for Polishing Deburring” will be denominated UBP, and similar nomenclatures were chosen for the other conditions, as presented in Table 1. The results were presented as the mean values and standard deviations. For the statistical analysis, a full factorial design was applied, considering a 95% confidence interval. The samples have two levels—trial and replica—and deburring has two levels—prior deburring and after deburring. Only 4 min of polishing were considered, and the machined length represented all of the 12 slots. For these results, analysis of variance (ANOVA) was performed for the single variables and also 2-way and 3-way interactions. For better visualization, the Pareto charts will be presented along with the ANOVA results.

## 3. Results and Discussions

### 3.1. Influence of Polishing Time on Burr Reduction

**Figure 3:**
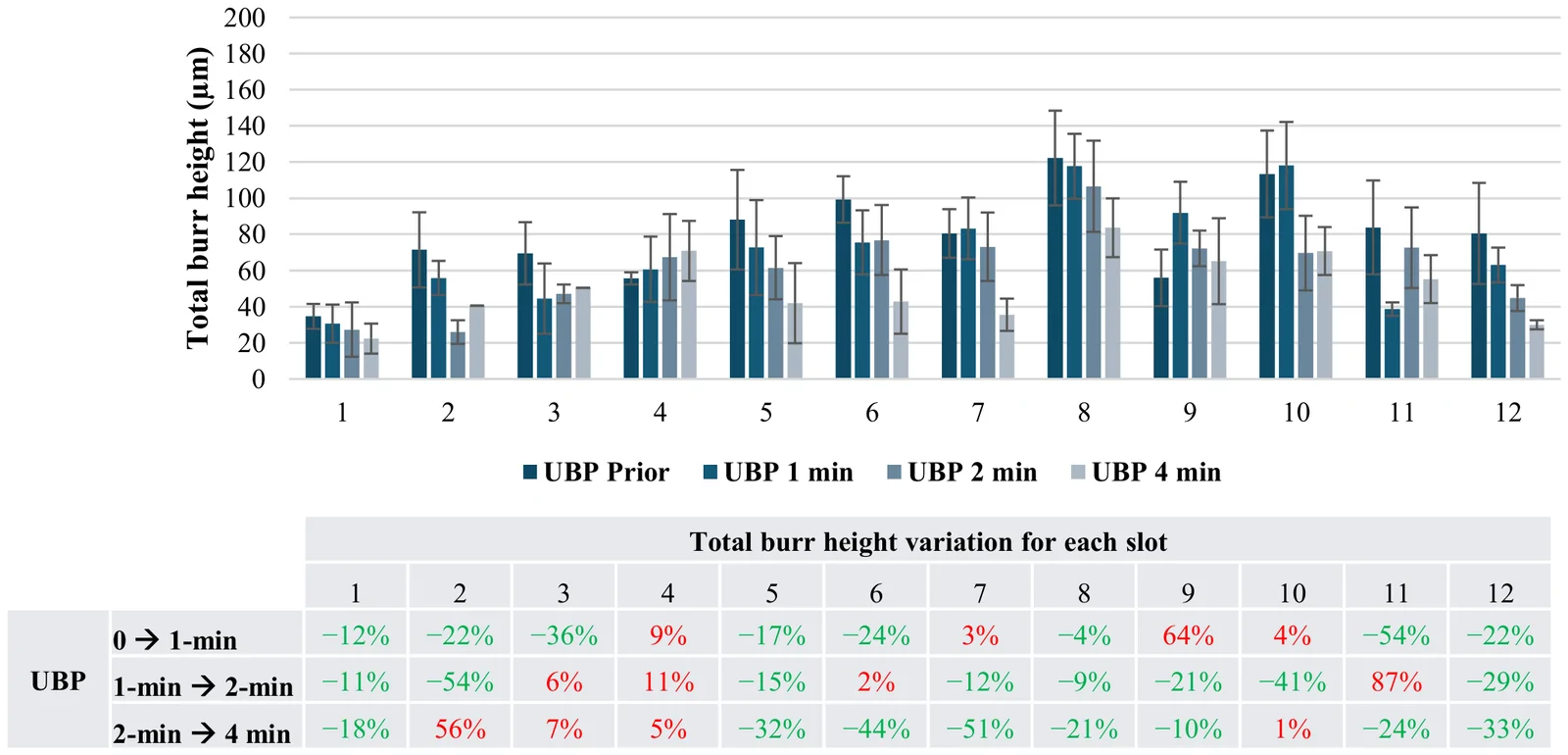
Total burr height over time for up-milling side. Error bars represent standard deviation.

**Figure 4:**
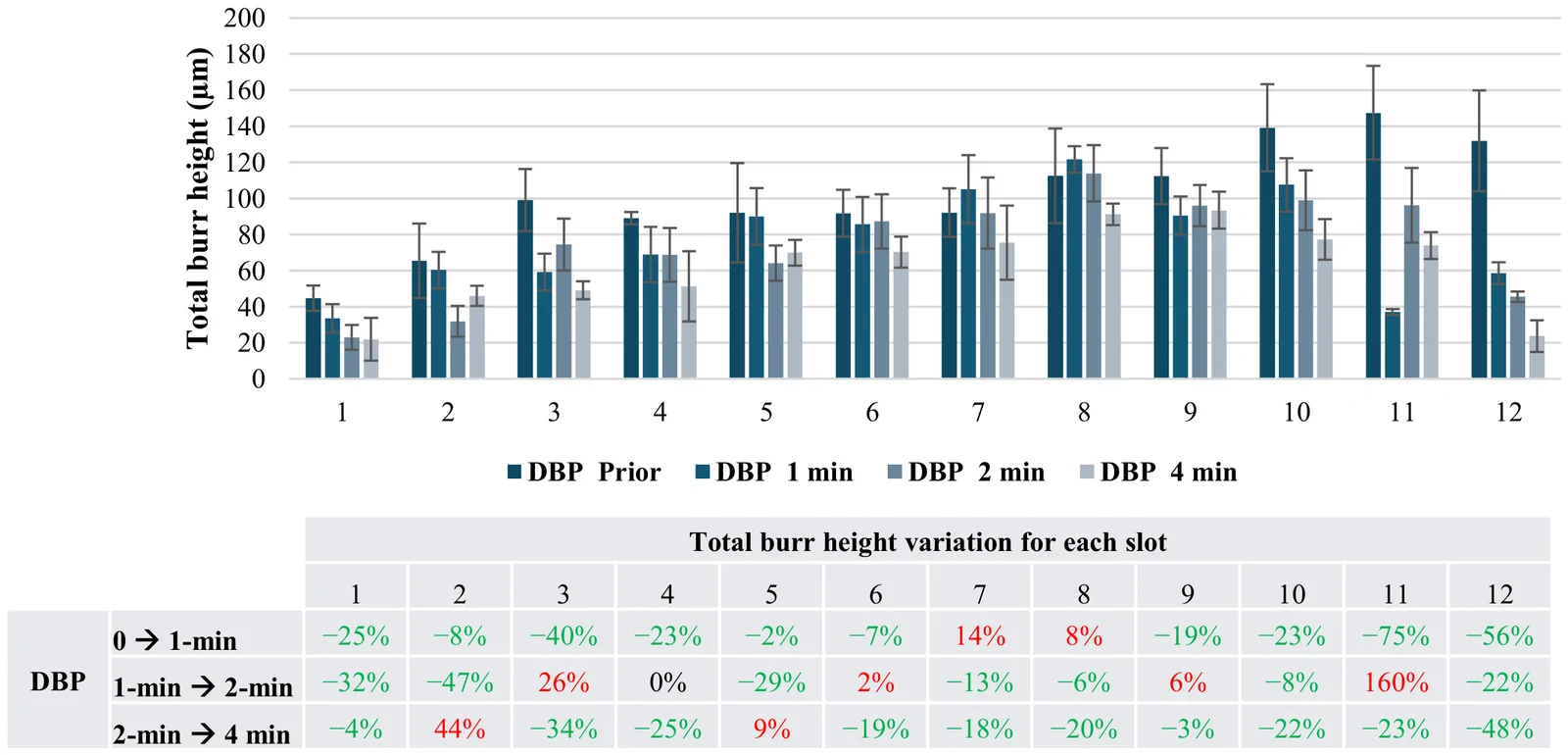
Total burr height over time for down-milling side. Error bars represent standard deviation.

In the literature, it has been reported that polishing can be efficient for improving the surface quality of micro-tapered holes [38], removing micro-milling tool marks [39], and eliminating surface defects from pulse compression gratings [40]. However, there is a lack of reference works on the use of this method to remove burrs from micro slots. Thus, prior to comparing the two deburring methods, there was interest in analyzing the efficiency of polishing for removing burrs from the slots of In718 produced by micro milling. As mentioned in Table 1, three deburring polishing times were considered (1 min, 2 min, and 4 min), and the total burr height after each one is shown in Figure 3 and Figure 4 for up-milling and down-milling sides, respectively. The results are shown for each one of the twelve slots, and the total burr height prior to the deburring process is also shown. One notes that for the up-milling side (Figure 3), slots 1, 5 to 8 and 12 presented a reduction tendency in total burr height with increasing the polishing time. This tendency, however, was less present on the down-milling side (Figure 4), being observed only for slots 1, 10 and 12.

**Figure 5:**
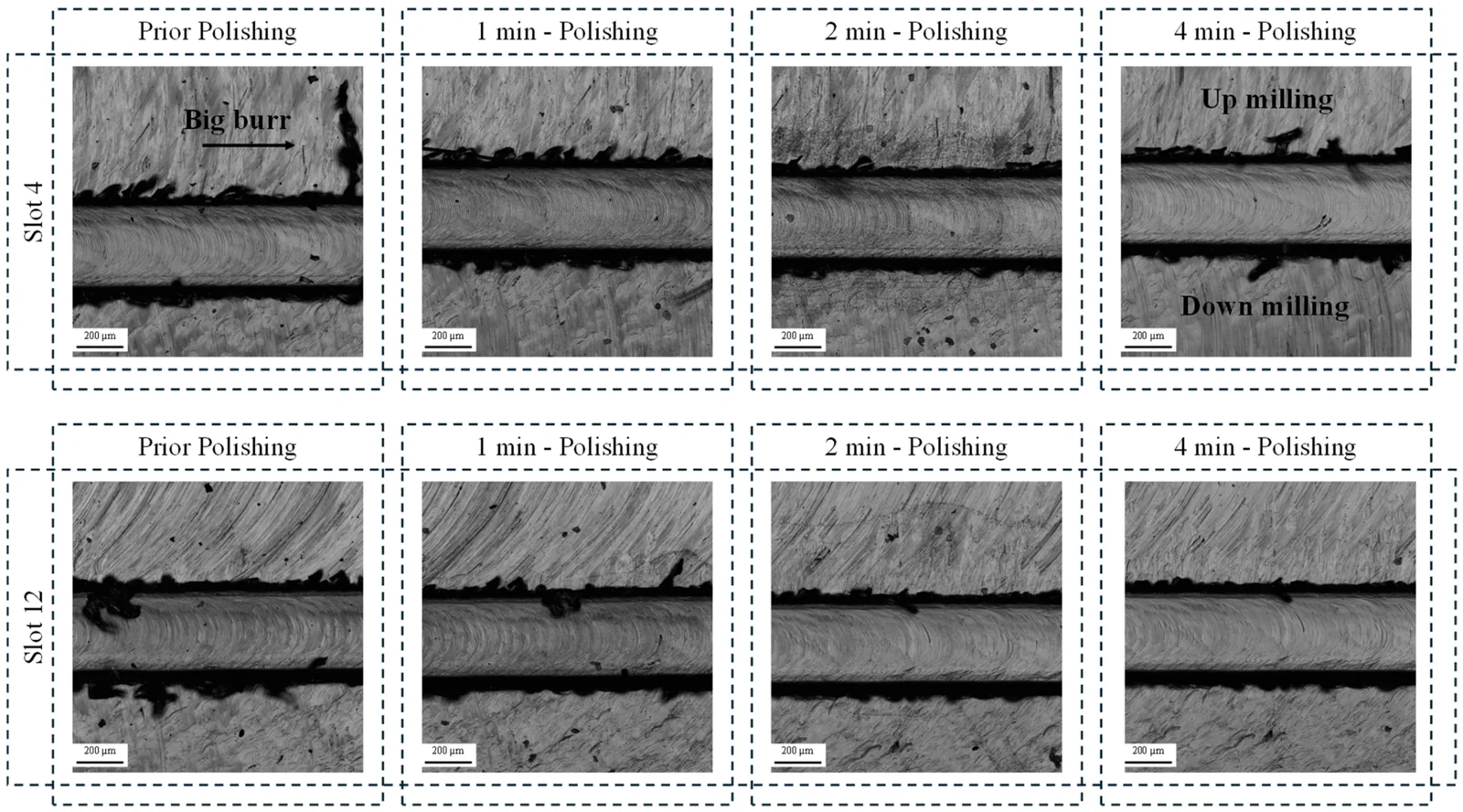
Details of burr’s evolution with polishing deburring time.

The increase in total burr height with polishing time was also observed for some results (e.g., slot 4—Figure 3). High geometric variations and distinct shapes of the burrs must be considered, and the material’s ductility as well, which contributes to both plastic deformation with no burr removal and high variability within measurements. To exemplify such effects, images of the burrs from slot 4 and 12 after deburring with different polishing times are presented in Figure 5. One notes that, for slot 12, burr removal occurred mainly in the first minute of deburring. When comparing the images from 2 and 4 min, the burr seems wider, although having lower height, which might indicate that not only was the burr removed, but it was also plastically deformed or displaced, with no necessarily mass reduction.

**Figure 6:**
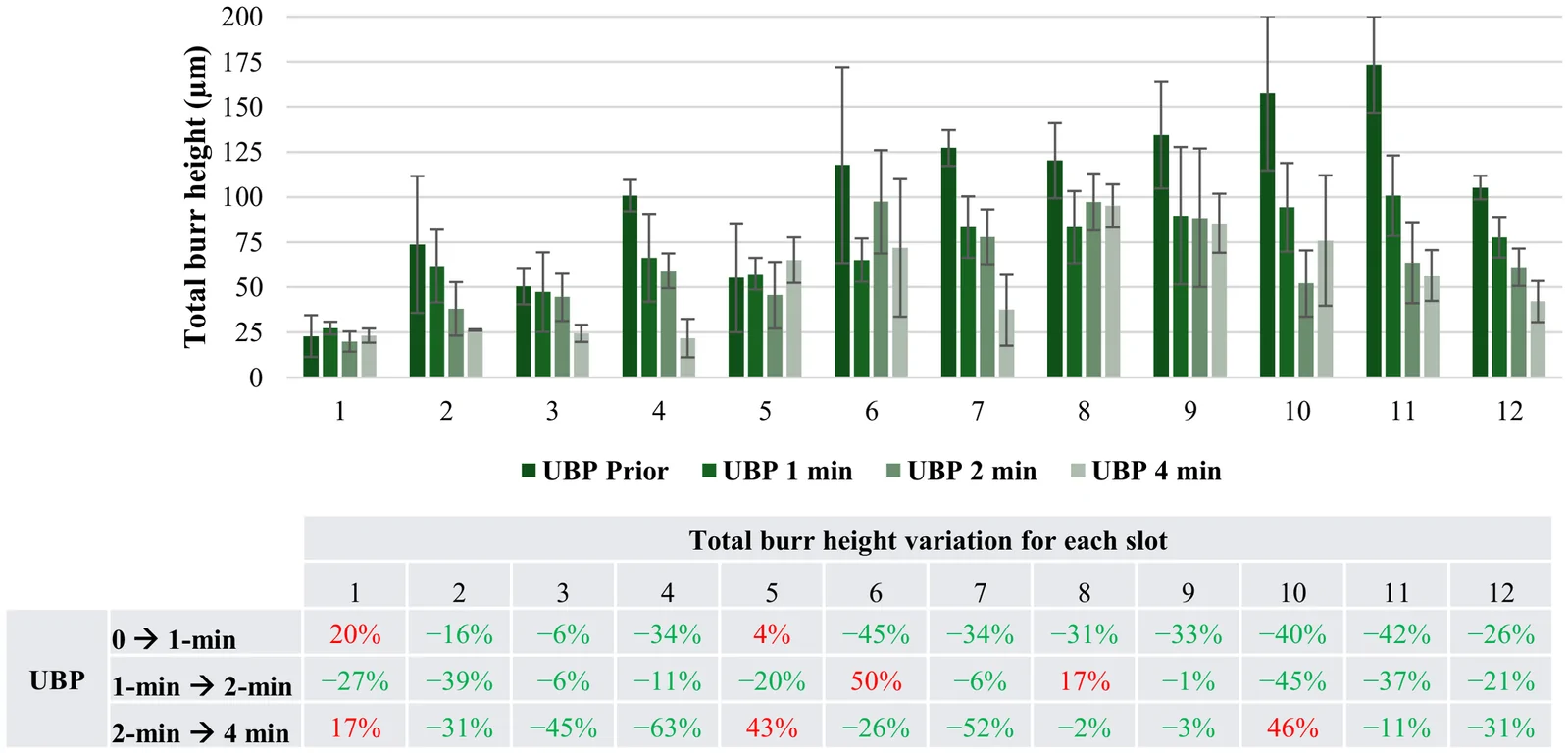
Total burr height over time for up-milling side—replica. Error bars represent standard deviation.

**Figure 7:**
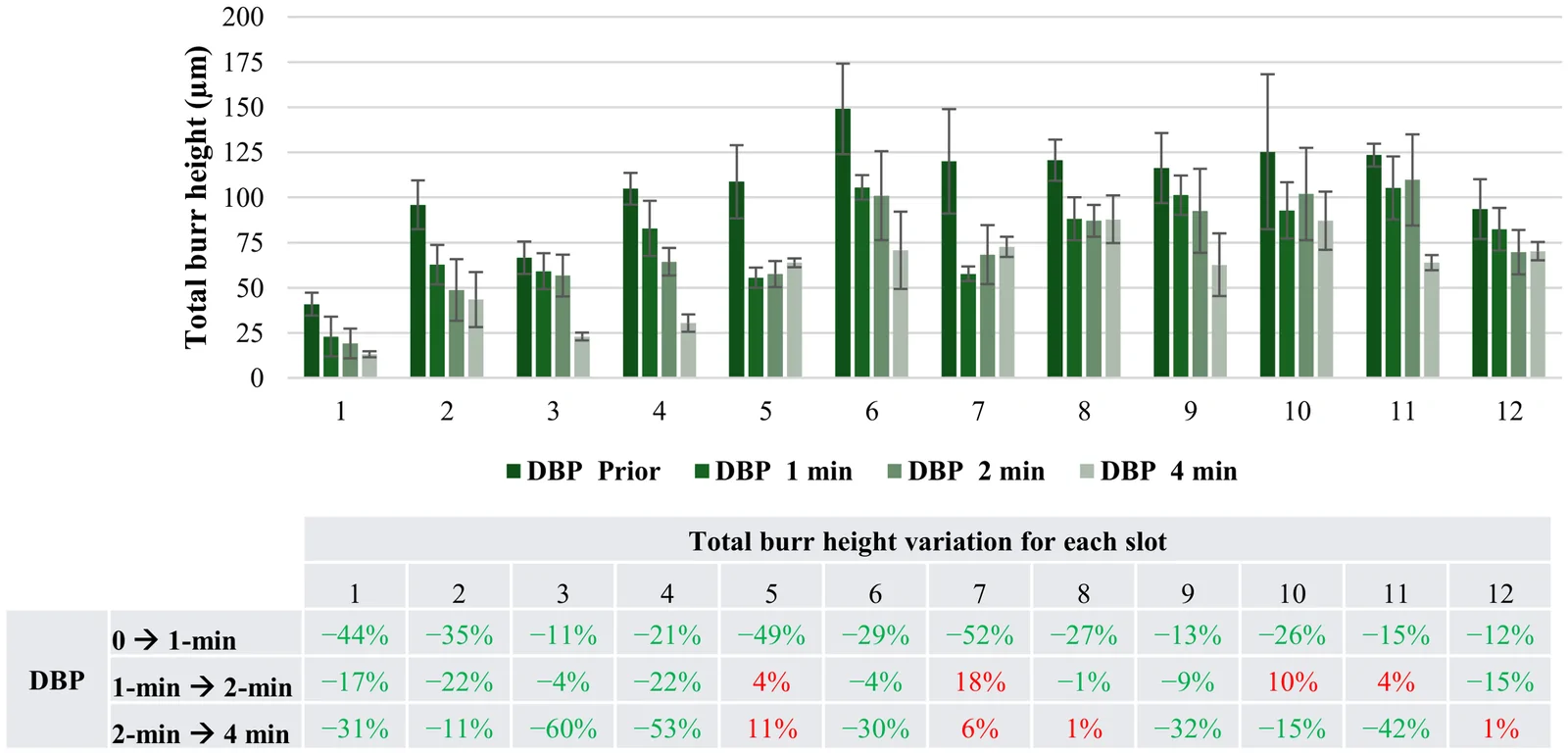
Total burr height over time for down-milling side—replica. Error bars represent standard deviation.

A similar plot can be observed for the replicas in Figure 6 and Figure 7. For the up-milling side, a clear relation of total burr’s height reduction over polishing time can be noticed for slots 2, 4, 7, 11, and 12. For the down-milling side, this behavior is obvious for slots 1, 2, 4, 6, and 9. It can also be noticed that, even after 4 min of deburring, the burr still presents a significant height compared to the target slot’s depth (a_p_ = 40 µm). According to Marinescu et al. [41], the abrasive action of polishing is gentle and presents a low material removal rate. This process is commonly used when the geometry of the part is already very close to the final geometry, so that its objective is usually modifying the surface texture of the part, allowing it to obtain a mirrored and reflective surface without significant dimensional alterations. Therefore, the fact that polishing was unable to completely remove the burrs is associated with its low material removal rate, a result of its inherent three-body abrasive mechanism which favors micro plowing and micro indentation mechanisms over micro cutting, which increases plastic deformation with no material removal [42].

**Figure 8:**
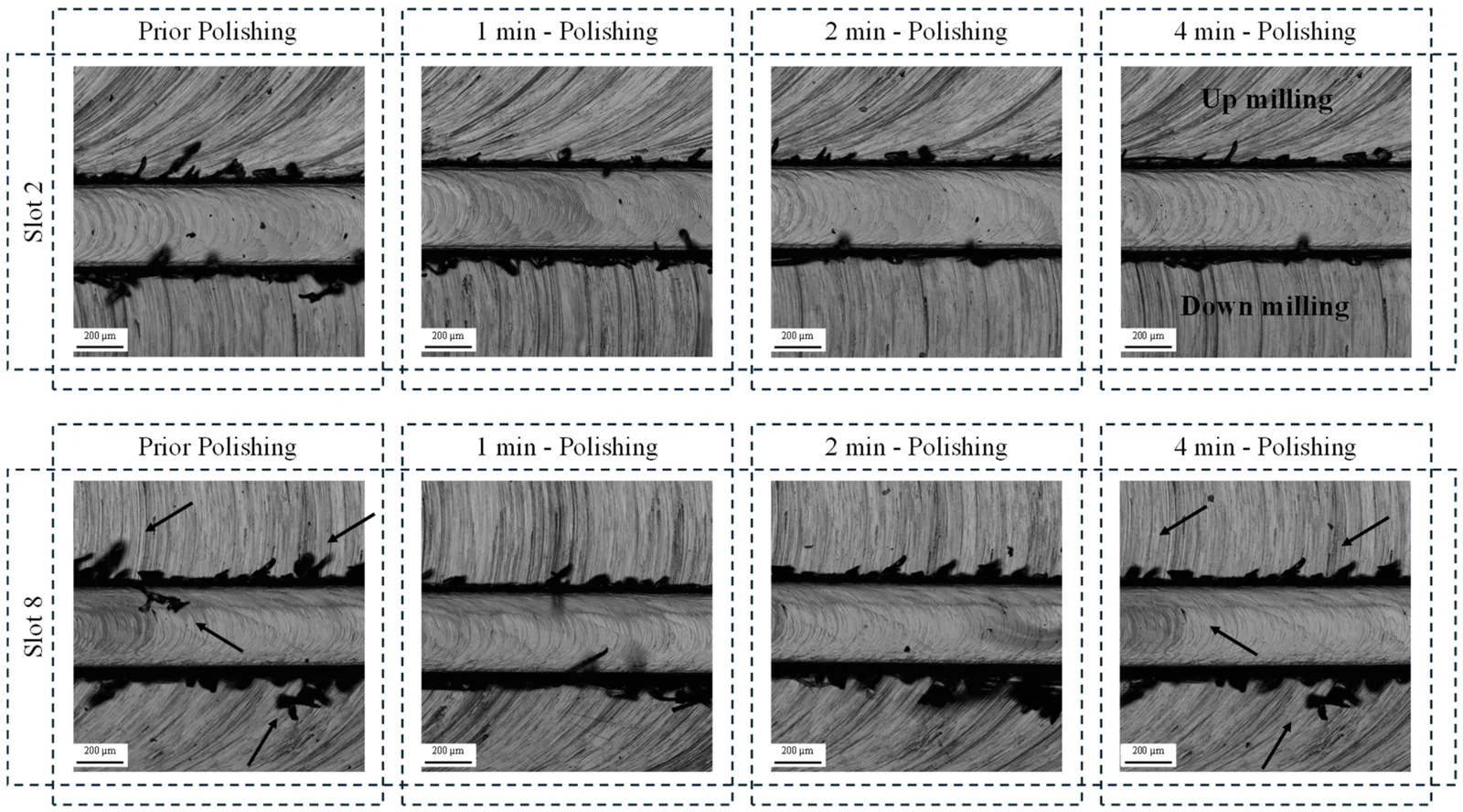
Details of burr’s evolution with polishing deburring time—replica.

Representative micrographs of the burr morphologies observed under different deburring conditions are shown in Figure 8. As can be noticed, a clear decrease in protuberant burrs was observed over the deburring time for slot 2. For slot 8, on the other hand, larger burrs were removed; however, after 4 min of deburring, observation of the same region (indicated by the black arrows) shows that the overall burr morphology remained similar. The presented micrographs are representative examples of the observed burr morphologies and are intended to support the qualitative discussion of burr morphology. The quantitative analyses, in contrast, were performed using the complete experimental dataset.

**Figure 9:**
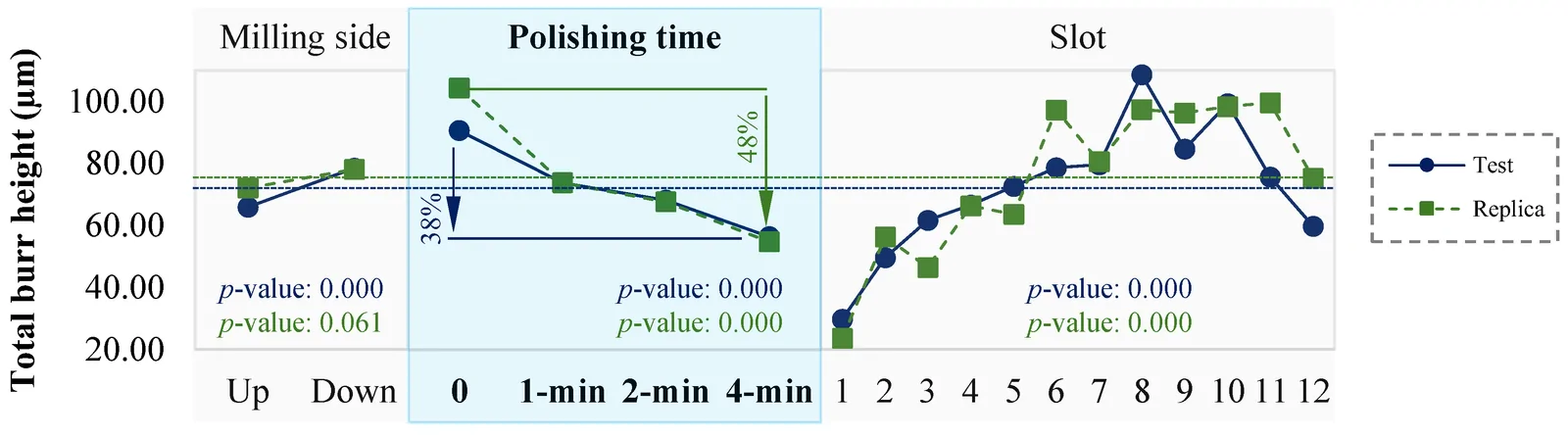
Main effects for total burr height polishing results. Dashed lines indicate the overall mean.

Thus, the effect of polishing deburring time on total burr height and its qualitative aspect indicates that polishing has limitations in removing burrs from micro-milled slots in In718. To better visualize the effect of polishing time, an analysis of variance (ANOVA) was performed, and the results for main effects are shown in Figure 9. As can be noticed, 4 min polishing time promoted the greatest reduction in total burr height: 38% and 48% in general for the test and replica, respectively. Nevertheless, as previously discussed, reductions in total burr height may not be due to material removal only, but also a consequence of burr’s displacement by plastic deformation.

### 3.2. Comparison Between Polishing and Sanding Methods

**Figure 10:**
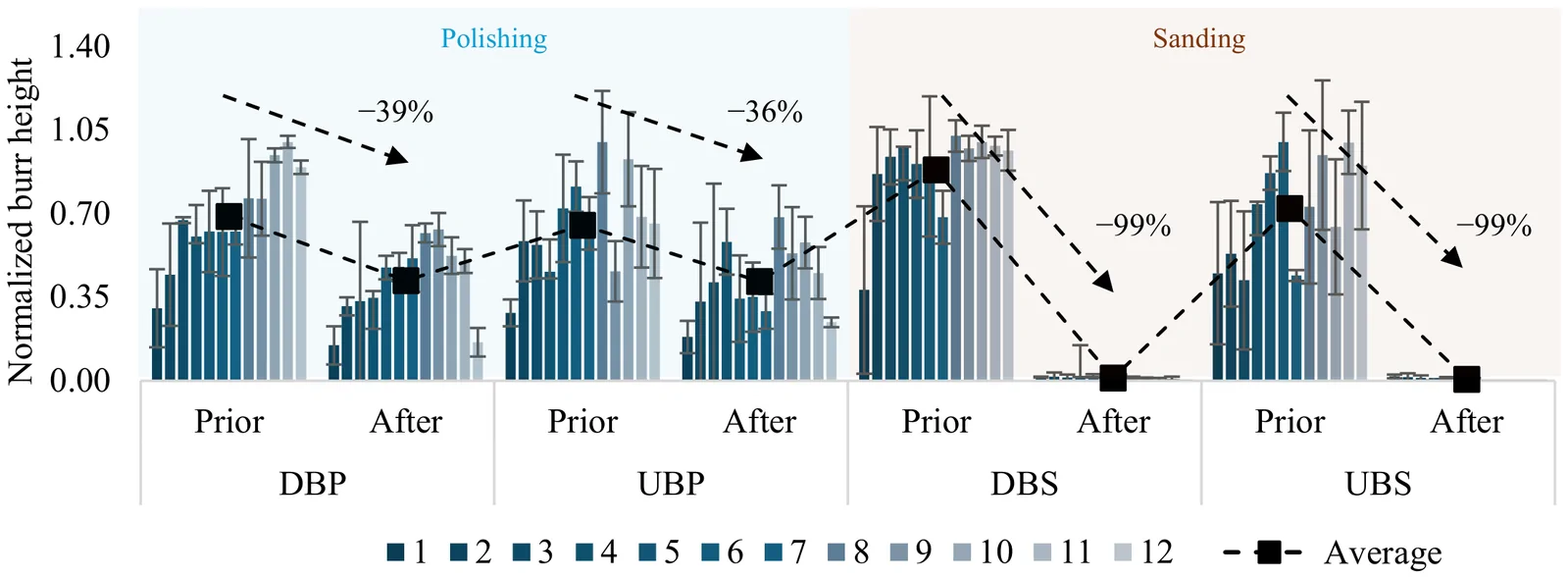
Burr height reduction after deburring by polishing and sanding. Error bars represent standard deviation.

**Figure 11:**
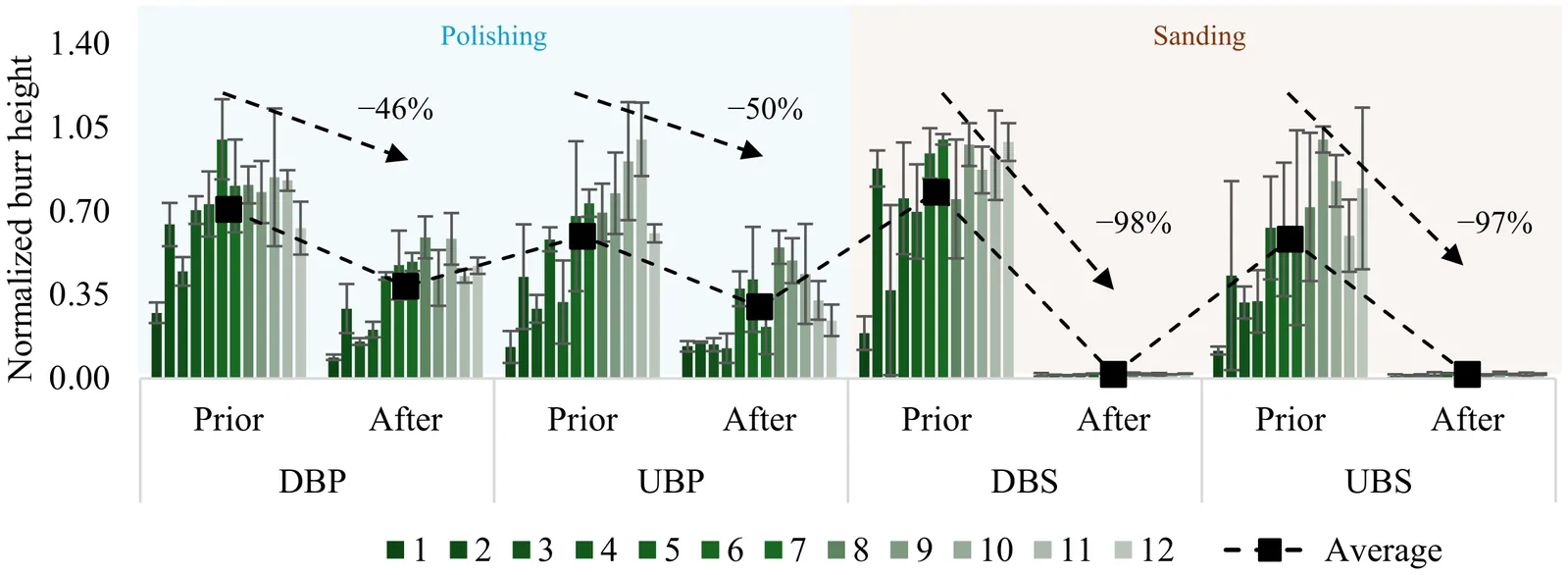
Burr height reduction after deburring by polishing and sanding—replica. Error bars represent standard deviation.

The burr heights after polishing for 4 min and with the 30 s sanding will now be compared. The 4 min polishing was considered for the comparison with sanding since it presented the best performance in terms of reducing the total height of burrs. In Figure 10, one can observe the results from the trials, and in Figure 11, from replicas. The results include the main average for each group, and the values of total burr height were normalized to facilitate comparisons. As observed, the burr height decreases greatly after the deburring by sanding, highlighting its efficiency for reducing the total height of burrs. Additionally, it is known that the milling strategy and parameters such as feed per tooth and depth of cut can have a significant effect on burr formation [43]. In this context, one notes from Figure 10 and Figure 11 that up-milling burrs presented a general average height lower than down-milling ones, which is consistent with the results of Fang et al. [31] in micro milling of Inconel718, and with the findings on other difficult-to-cut materials, including AISI 304 stainless steel [24] and Ti-6Al-4V superalloy [44]. Nevertheless, the specific side with the largest burrs of milled micro slots can also vary during the process depending on micro tool wear as observed by Ziberov et al. [45].

**Figure 12:**
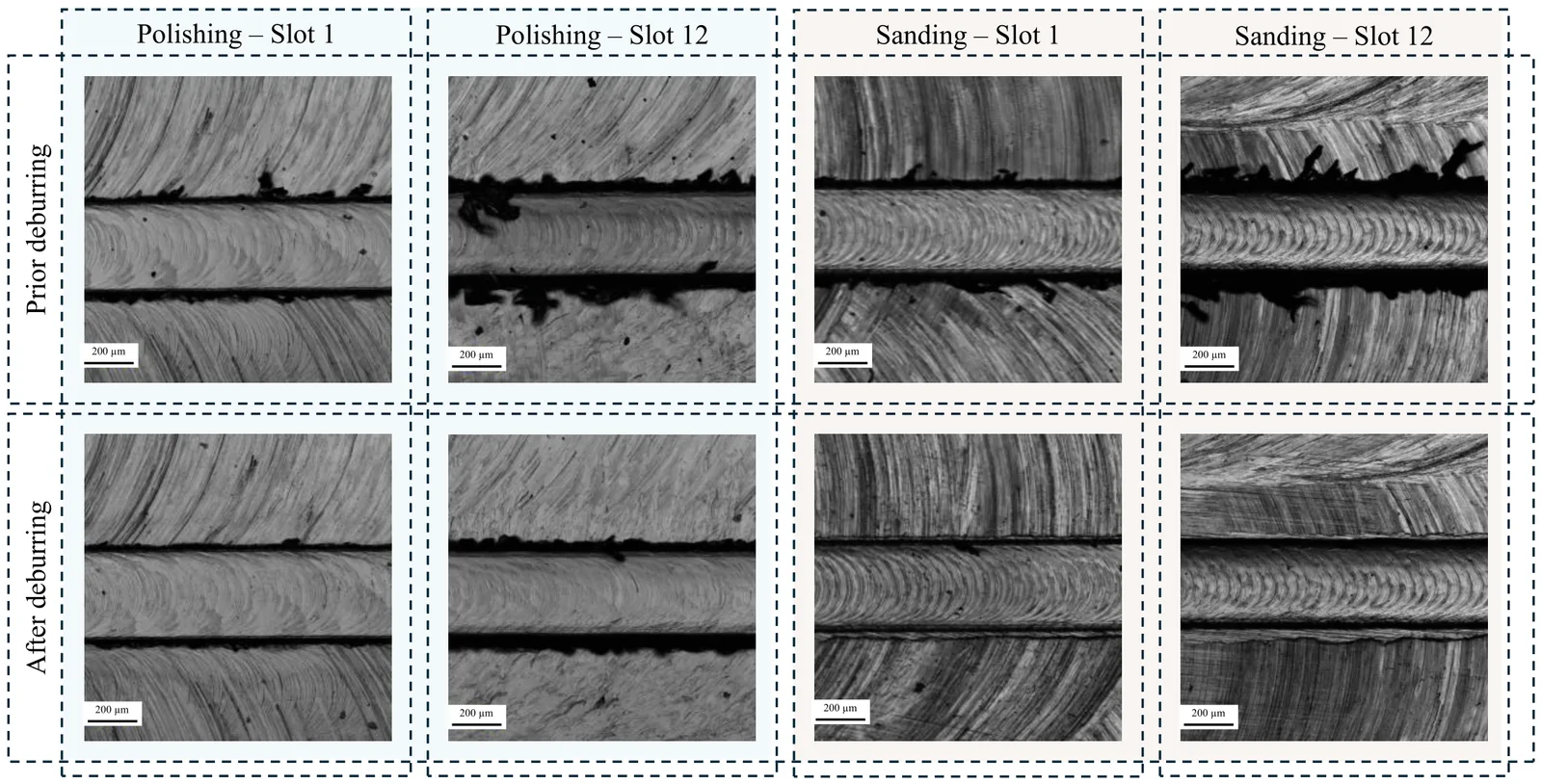
Burr characteristics prior to and after deburring by polishing and sanding.

For the burr height after deburring, it should be noted in Figure 10 and Figure 11 that the standard deviation values for polishing results (DBP and UBP) were significantly higher compared to the sanding results (DBS and UBS), which is associated with the higher material removal in sanding deburring, which practically removed the burrs relative to polishing. Nevertheless, Figure 12 shows the burrs of slots 1 and 12 for each deburring process for better comparison.

**Figure 13:**
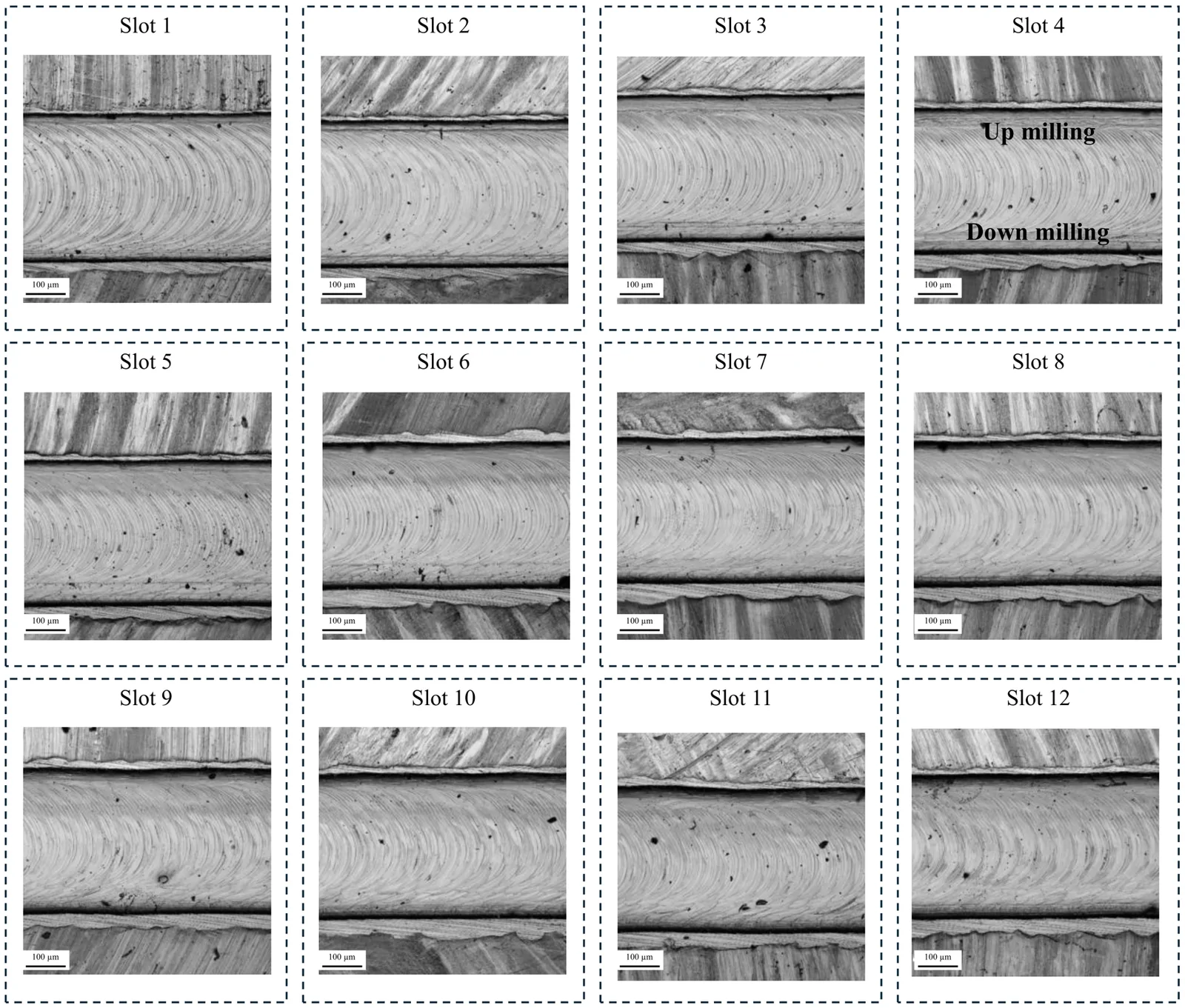
Resulting burrs after sanding.

It can be noticed that polishing reduced burrs but, as prior to deburring, it still presented irregular and relatively high height, as evidenced by the darker color in the microscopy images. Sanding, on the other hand, resulted in a more regular surface burr, with flat characteristics and small height values, allowing the surface to be observed more clearly by microscopy. To further visualize this effect, the details of the resulting burrs after sanding deburring are presented in Figure 13.

**Figure 14:**
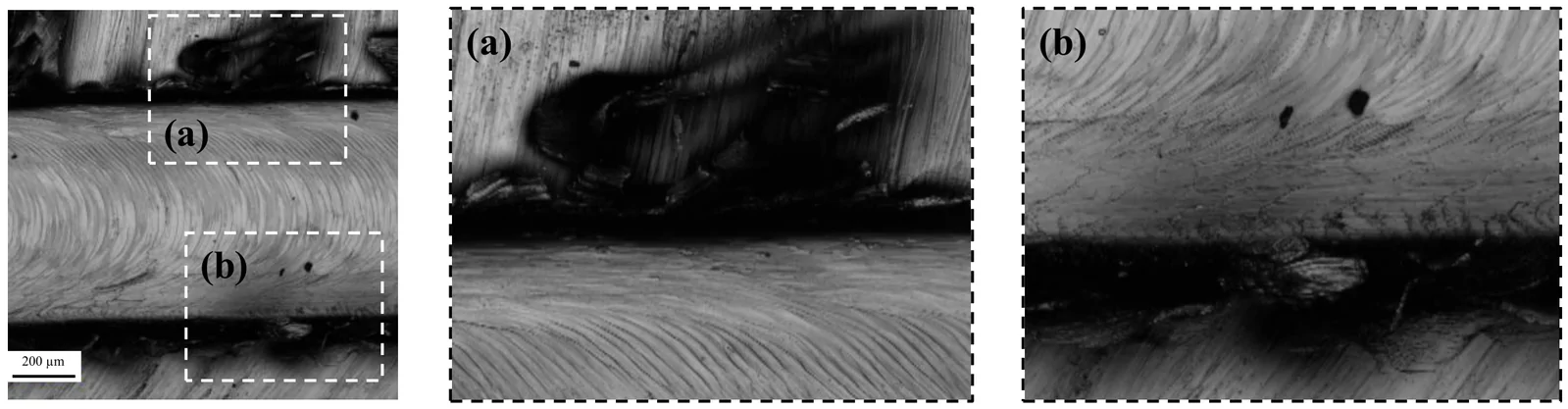
Details of a burr with high heights after polishing and deburring; (**a**) up-milling side, (**b**) down-milling side.

It can be noticed from Figure 13 that burrs are in fact flat, which is evidence that sanding deburring presented a significant compression effect by plastic deformation besides typical abrasive micro mechanisms. Additionally, slots with higher initial burr’s height (prior to deburring) presented, in general, a higher width of the flat burr’s area, suggesting no direct relationship between initial burr’s height and actual material removal for the sanding deburring process. Similar analysis was not performed for polishing deburring due to the impossibility of obtaining a focused image, as demonstrated in Figure 14. Added to this, with this magnification, it was not possible to properly represent all the burr area in the image.

Therefore, the results show that the post-processing by sanding is significantly more effective than polishing in terms of reducing the total height of burrs. Additionally, its efficiency in terms of processing time and abrasive contact length demands is also higher; sanding was able to reduce the total height of burrs by 97–99% after 30 s and 18.85 m of contact length, while polishing reduced it by only up to 48% after 4 min and 38.4 m of contact length. When comparable contact lengths are compared (as for 2 min polishing), both effectiveness and efficiency of sanding over polishing become even more evident. Lastly, it is worth mentioning that the differences between abrasive grits may also play a role: while sanding employed 7 µm silicon carbide (SiC) grits, polishing was performed with 3 µm diamond grits as specified in Figure 1. The larger grit size likely contributes to the material removal, which is already higher for the two-body abrasion mechanism involved in sanding. Greater abrasive hardness also enhances material removal by increasing the indentation capacity. However, silicon carbide is already significantly harder than the In718 (>>1.7 times); therefore, the substantially higher hardness of diamond grits has only a minor influence in this case [42], especially considering the three-body abrasion mechanism.

### 3.3. Impact of the Deburring Method on Surface Roughness

**Figure 15:**
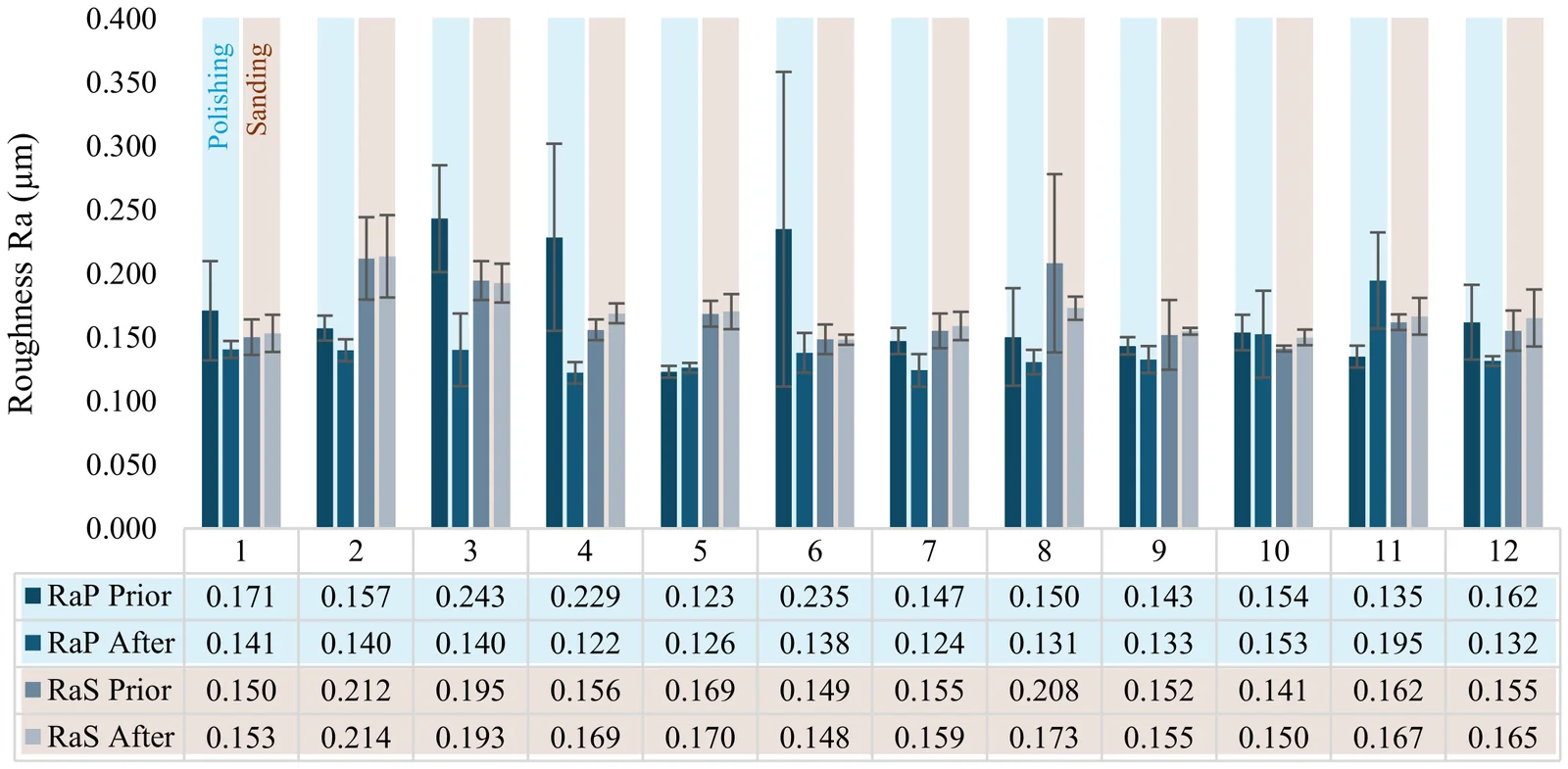
Slot’s surface roughness (Ra) after deburring by polishing and sanding. Error bars represent standard deviation.

**Figure 16:**
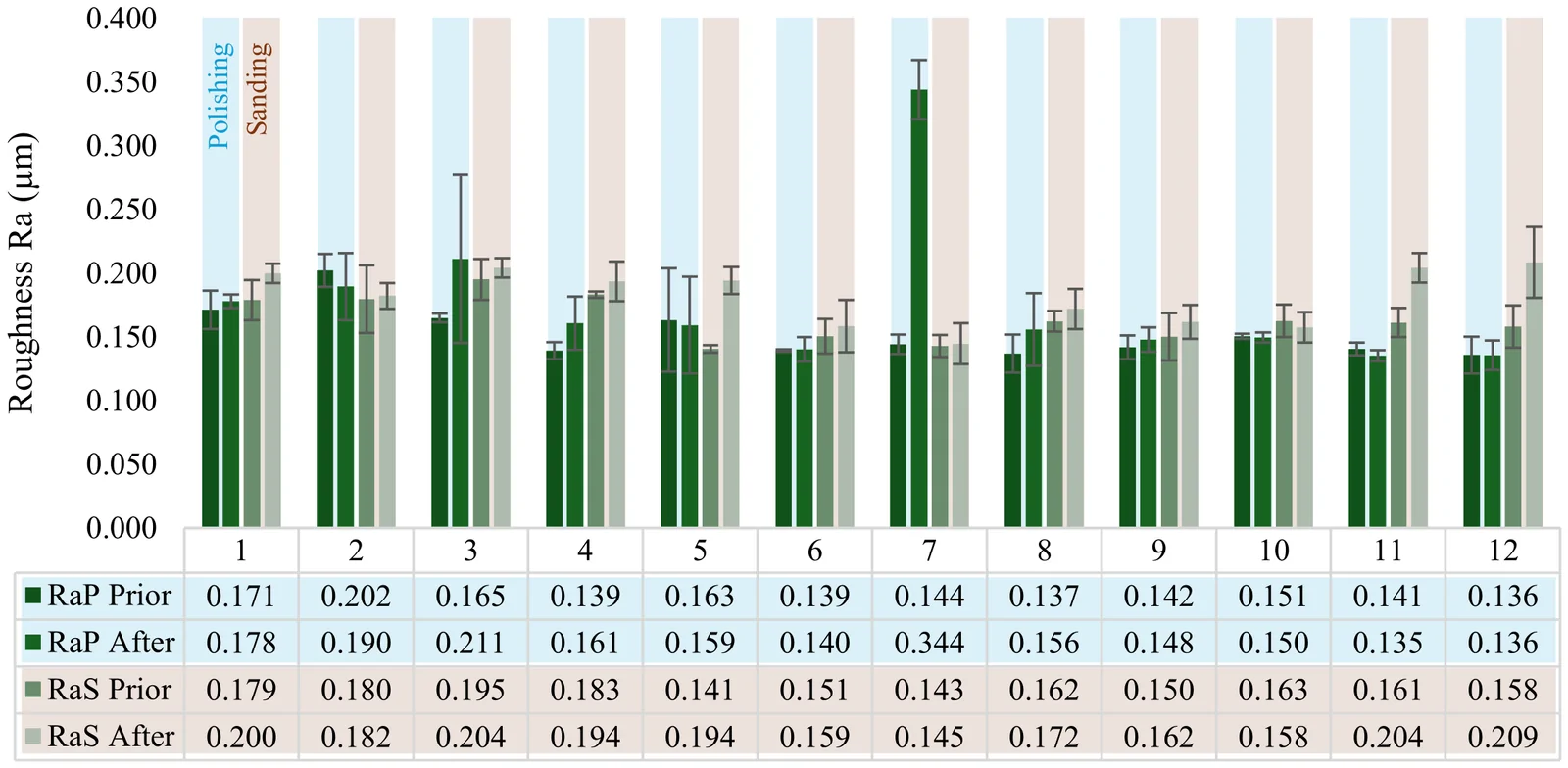
Slot’s surface roughness (Ra) after deburring by polishing and sanding—replica. Error bars represent standard deviation.

It is important that the deburring process is able to remove burrs without damaging the slot surface. Thus, the surface roughness Ra was analyzed and the results are shown in Figure 15 and Figure 16. In these graphs, the value prior to deburring is presented alongside the value after deburring for both methods, grouped by the slots (machined length). Overall, the roughness reduced after deburring by polishing, except for slots 5, 10 and 11 (first group of experiments) and slots 4, 7 and 8 (replica). As for the sanding, differences were minor, with some slots presenting higher roughness values after deburring, especially for the replica (Figure 16).

**Figure 17:**
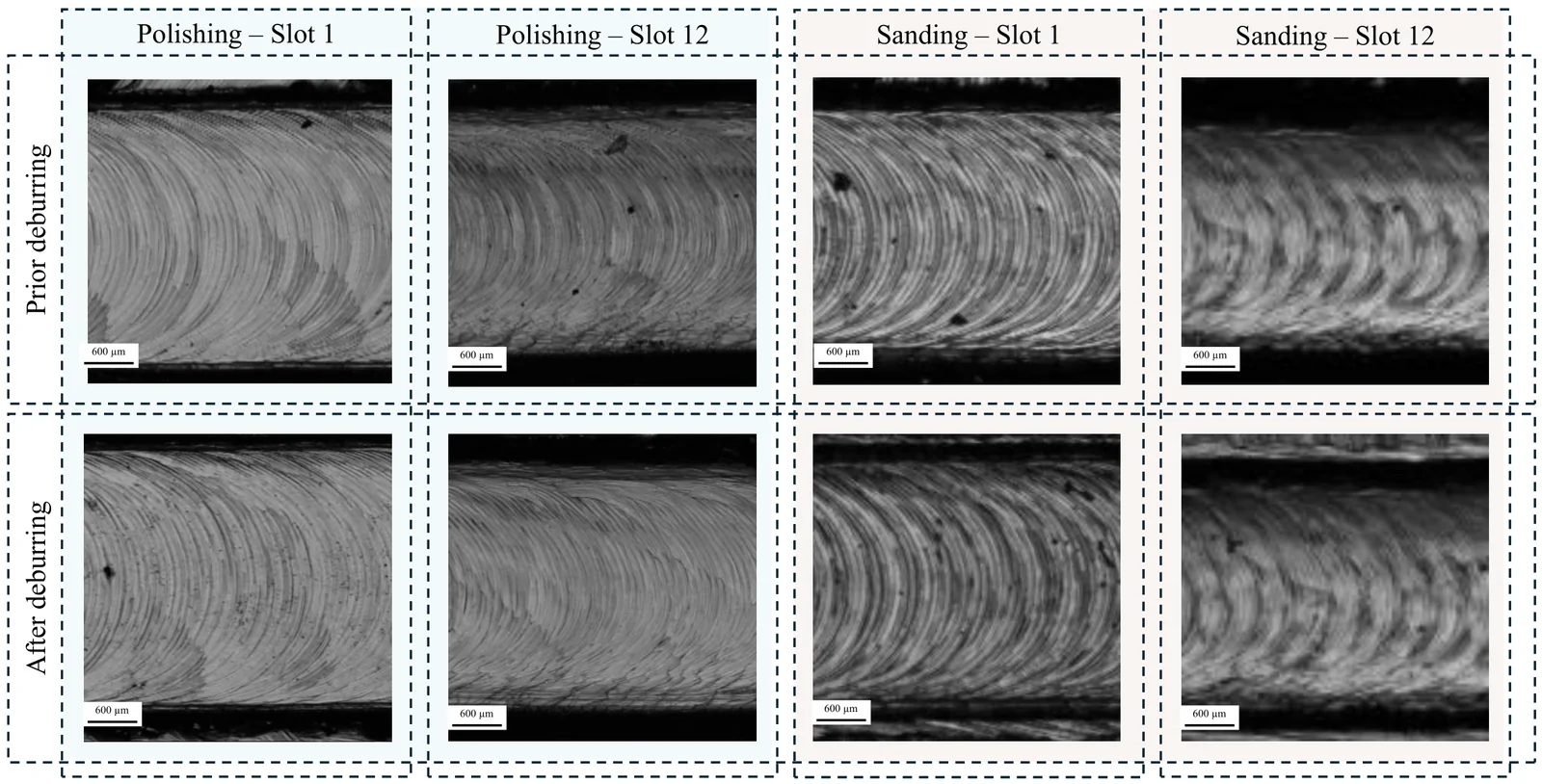
Detail of the surface prior to and after deburring by polishing and sanding.

The greatest improvement in roughness was observed in slot 6 for the trial, with a reduction of 0.097 μm on the Ra value. For polishing, the worst result was an increase of 0.200 μm (slot 7 replica), attributed to possible damage from a detached burr. For sanding, the highest increase was 0.054 μm on slot 5, and the most significant decrease was 0.005 μm for slot 10, both on the replica. Although the difference in values, the slot surface remained similar to that demonstrated in Figure 17. It is possible that, during sanding, fragments of burrs detached from the workpiece scrape the bottom surface of the micro slots, which is less likely to occur in polishing due to the lower material removal capacity.

Roughness is a common output to assess the quality of surfaces after machining; Fang et al. [31] obtained a roughness of 0.167 μm, a value close to the average value obtained in the present work, which resulted in 0.176 μm. Aslantas and Çiçek [10] analyzed the average roughness values for different lubrication conditions of the micro-milled Inconel 718 sample. The lowest roughness value obtained was for MQL micro milling, which resulted in 0.200 μm Ra, a value close to that obtained in this work, which was 0.176 μm. Wu et al. [46] used laser-induced plasma deburring (LPD) to remove burrs from micro channels on an aluminum alloy workpiece. It was found that the LPD process causes very small changes in the surface roughness, hardness and grain size of the micro slot side wall. This suggests that debris resulting from the deburring process is less harmful to the micro component’s surface in laser deburring than in abrasive deburring, even though the LPD is associated with higher costs and the necessity of using a sacrifice plate for the laser ablation.

### 3.4. Statistical Evaluation and Main Effects for Burr Heights

**Figure 18:**
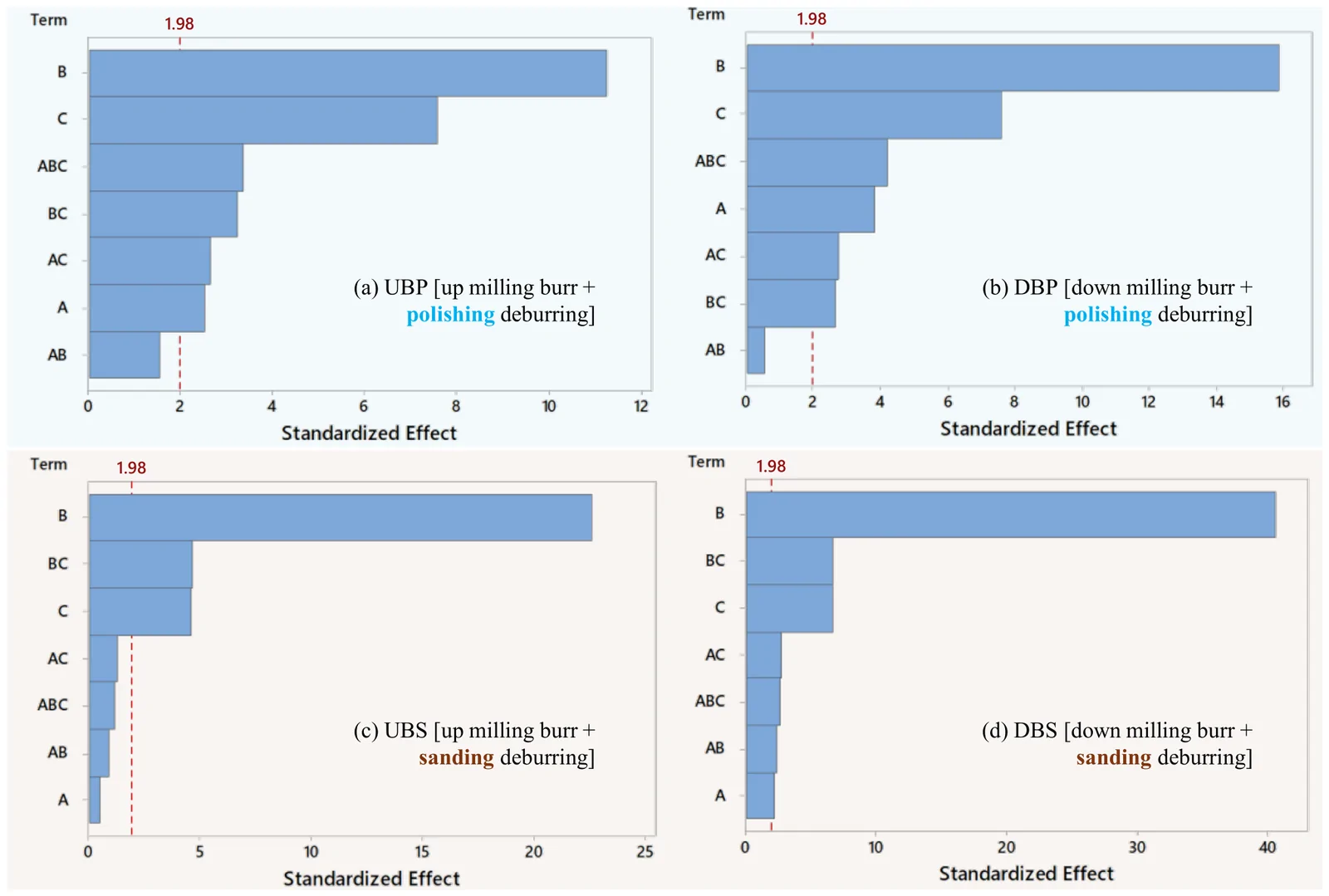
Pareto chart for standardized effects for burr with α = 0.05, A = sample, B = deburring and C = slot. (**a**) UBP, (**b**) DBP, (**c**) UBS and (**d**) DBS.

*p*-values and Pareto charts for the ANOVA results are shown in Table 2 and Figure 18, respectively. The factors and respective levels considered were sample (A-test and replica), deburring (B-prior and after), and slot (C-1–12, correlated to machining length). The analysis included main effects and all interactions. The responses were up- and down-milling burr’s height for polishing (UBP and DBP), and up- and down-milling burr’s height for sanding (UBS and DBS).

It can be noticed that deburring (B) and slot (C) were totally significant for all the responses, with *p*-values equal to zero. This result confirms the effect of deburring techniques on the burr heights. As for the factor slot (C), it should be emphasized that this factor was included in the statistical analysis to account for the individual burr height measurements obtained from each micro slot, rather than to directly represent the progressive evolution of tool wear. This approach enabled the comparison of the deburring response for each slot independently, allowing the statistical analysis to identify possible differences associated with the slot position during the deburring process. Such an evaluation is particularly relevant because the abrasive interaction may not be perfectly uniform over the entire workpiece, and slots located at different positions could experience distinct local contact conditions during polishing or sanding. Although machining length is commonly associated with progressive tool wear and burr formation, as reported in previous studies [4,47,48,49], the present work did not include direct measurements of tool wear. Therefore, the statistical significance of the slot factor should not be interpreted exclusively as evidence of tool wear progression, but rather as the combined effect of slot-to-slot variability, machining length, and possible positional influences during the deburring operation. Future studies including direct tool wear measurements are recommended to isolate the contribution of wear from the other factors investigated.

The factor sample (A) was expected to have a low influence on burr formation, since cutting parameters [48] and tool wear [49] are usually the main influencing factors. However, except for UBS, the sample factor presented a *p*-value < 0.05 for all other responses, indicating a significant difference between the two experimental trials under the same cutting conditions. Since only two sample levels were evaluated, this effect should not be interpreted as a generalizable effect of the sample factor, but rather as the variability between the two specific replicas, which may be associated with factors such as local material microstructure heterogeneity, as previously reported for In718 micro milling [35], and other uncontrolled experimental variations. Therefore, future studies should employ a larger number of replicas to improve statistical representativeness and allow a broader statistical generalization of this effect. Additionally, *p*-values for the interaction of sample with deburring (AB) and slot (AC) are higher, indicating a lesser effect on the results. The deburring and slot interaction (BC) remained significant for all the burrs evaluated.

In terms of effect on the responses, one notes from Figure 18 that deburring (B) possesses the main influence in burr’s height, irrespective of milling mode and deburring process. For polishing, the slot (C) was the second most influential factor, followed by the three-way interaction. For sanding, on the other hand, the interaction between deburring and slot (BC) presented the second most influential effect, which might have been caused by the higher removal capacity of the sanding process.

**Figure 19:**
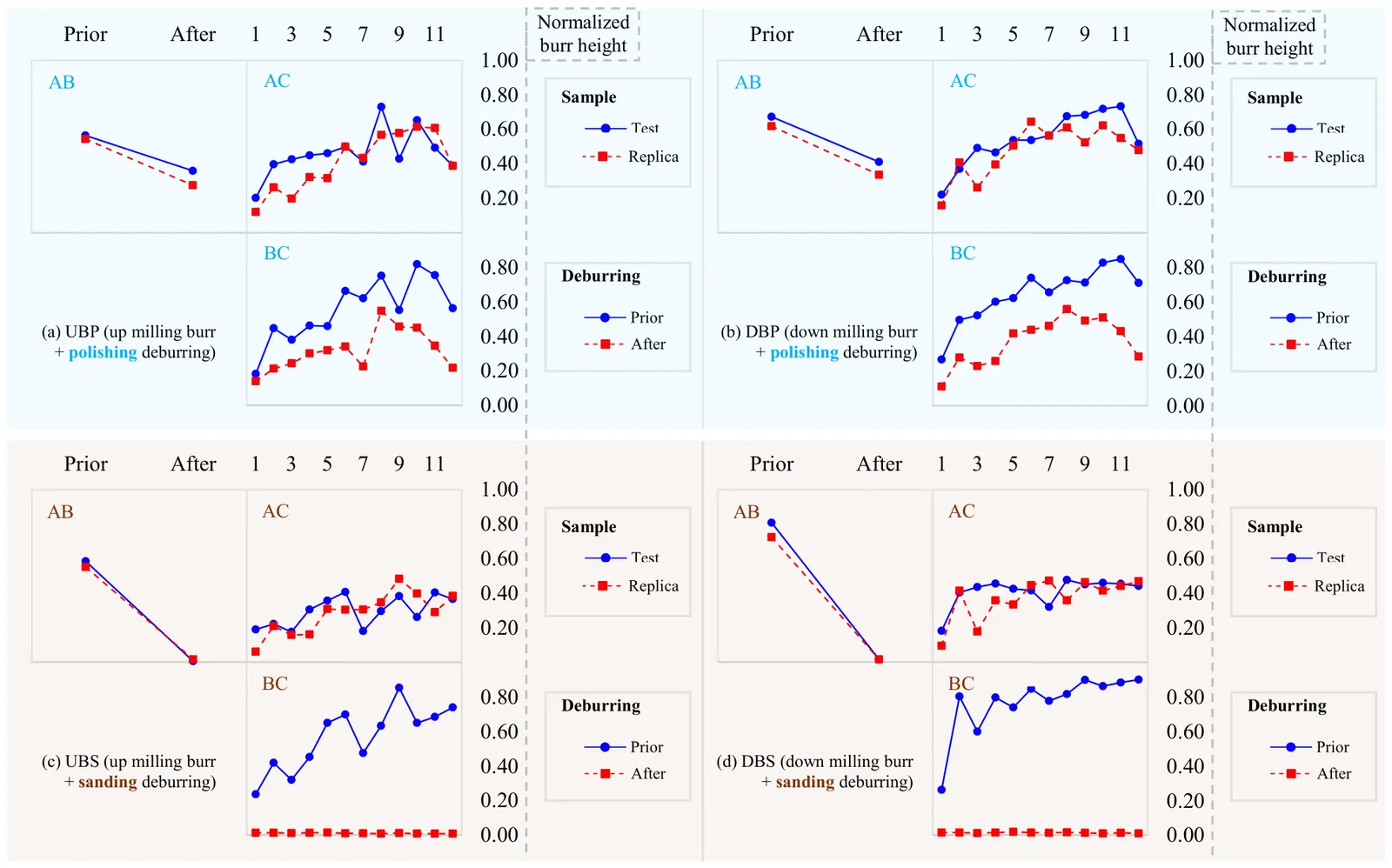
Interaction plots for burr height. (**a**) UBP, (**b**) DBP, (**c**) UBS, (**d**) DBS.

The interaction plots are shown in Figure 19, which puts in evidence the trends discussed in Section 3.3 and Section 3.4. For instance, when focusing on the interaction between sample and deburring (AB), it can be noticed that after the deburring process, all burr’s height reduced in a similar way for both test and replica, with a higher slope for sanding due to the higher material removal capacity as discussed earlier. Analyzing the interaction between the sample and slot (AC), the curves for test and replica also have similar trends, although not the exact same behavior. Comparing the curves in the interaction between deburring and slot (BC), the burr reduction is clear for all slots and all the milling sides, especially after sanding, which reduced burr’s height near to zero.

### 3.5. Statistical Evaluation and Main Effects for Roughness

**Figure 20:**
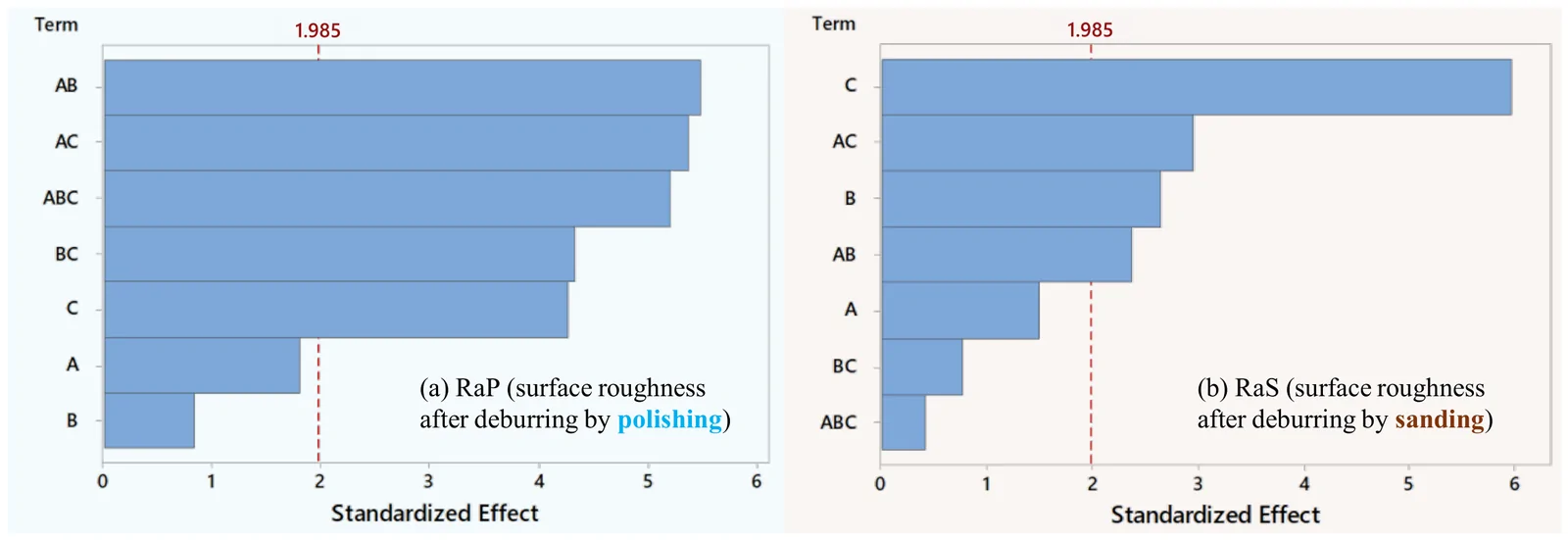
Pareto chart for standardized effects for Ra with α = 0.05, A = sample, B = deburring and C = slot. (**a**) RaP. (**b**) RaS.

Similarly, the analysis of variance was also performed for roughness results and the *p*-values and Pareto charts are shown in Table 3 and Figure 20, respectively. These results aim to statistically quantify the impact of each deburring process on the micro-slot surface finishing (Ra parameter). One note from Table 3 is that *p*-values for the factor samples (A) were higher than 0.05, which indicates no statistically significant differences in micro-slot finishing between the first group of experiments (test) and the second one (Replica). For the factor deburring (B), no statistical significance was observed for the polishing deburring (RaP); for the sanding deburring (RaS), on the other hand, a *p*-value of 0.010 was found, thereby indicating a significant effect on surface finishing of micro slots. The factor slot (C) presented statistical significance for both deburring processes.

**Figure 21:**
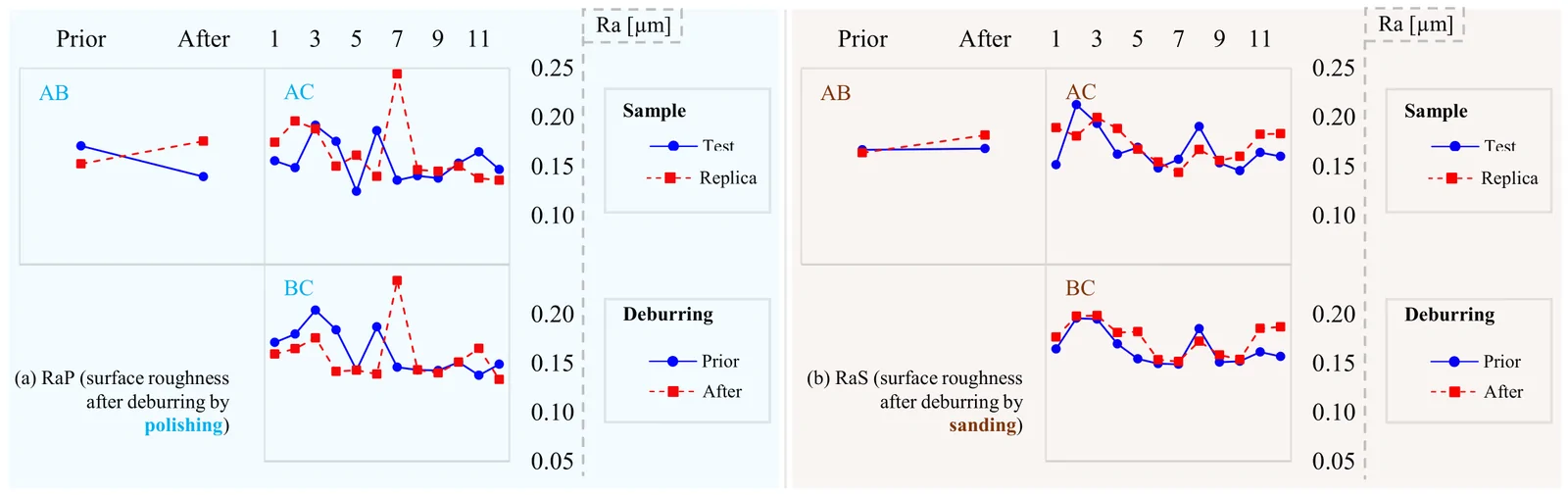
Interaction plots for surface roughness after deburring by polishing (**a**) and sanding (**b**).

From the interaction plots (Figure 21), one notes that the interaction between sample and deburring (AB) presented opposite trends for each deburring process: in the case of polishing, deburring in general decreased Ra for test but increased for replica; for sanding, it slightly increased for both cases. However, it is worth mentioning that the increase in mean average in Ra after polishing deburring for replica is associated with an outlier (slot 7), which presented a 61% increase in Ra, significantly contributing to increasing the mean average. In exception to this outlier, polishing deburring reduced Ra values for most slots, as shown in the interaction curves between deburring and slot factors (BC), with a maximum reduction of 21% (slot 6). Conversely, sanding deburring mostly increased Ra values of the micro slot, with a maximum increase of 19% (slot 12). One hypothesis to explain this result may be associated with the higher material removal rate of the sanding process, which may increase the amount of debris inside the micro slots that can damage the machined surface, thus increasing Ra values.

In summary, the deburring processes investigated exhibit distinct and, in some aspects, complementary effects on burr morphology and micro slot surface integrity. Polishing deburring, although contributing to reductions in Ra for most conditions, demonstrated a limited capability in effectively reducing burr height and in promoting substantial changes in their morphological characteristics. In contrast, sanding deburring proved to be highly effective in reducing burr height, practically eliminating it under the tested conditions and within shorter processing times, while also modifying burr morphology toward a predominantly flat surface profile. However, this enhanced material removal capability is accompanied by a tendency to increase surface roughness, with Ra increments reaching up to 19% in the present study. Therefore, the selection of an appropriate deburring strategy should consider multiple factors, including surface roughness requirements, desired burr morphology, and processing time constraints. Additionally, a combined approach incorporating sanding followed by polishing may be a proposed direction for future work to investigate combined effects, albeit at the expense of increased post-processing time and cost.

## 4. Conclusions

After evaluating different abrasive deburring methods for slots micro milled on Inconel 718, the following conclusions can be drawn:

- Burr formation in micro milling of Inconel 718 is significant, with burr heights reaching the same order of magnitude as the axial depth of cut. Additionally, down milling consistently produces higher burrs than up milling, indicating a strong influence of cutting kinematics on burr generation.
- Polishing deburring exhibits limited effectiveness in burr removal. Due to the high mechanical strength of Inconel 718 combined with the inherently low material removal rate of the polishing process, burrs are predominantly plastically deformed or only slightly reduced in height, rather than effectively eliminated.
- Sanding deburring demonstrates high effectiveness in reducing the total height of the burrs (up to 99% reduction) under the investigated conditions. Furthermore, this process induced significant morphological changes, with burrs assuming a predominantly flat surface profile as a result of the more aggressive material removal mechanism.
- The deburring methods present a trade-off between burr height reduction and surface integrity. Polishing showed limited effectiveness in burr height reduction but generally reduced Ra (up to 21%), whereas sanding produced flat morphologies (burrs with nearly zero height), at the expense of increasing Ra by up to 19%. Therefore, the selection of the deburring method should consider both surface roughness requirements and burr characteristics.

## Figures and Tables

**Table 1:** Output variables nomenclature.

Test	Sample	Deburring	Time	Burr Measured	Nomenclature for Burr Results	Nomenclature for Ra Results
1	Trial	Polishing	1, 2 and 4 min	Up milling	UBP	RaP
				Down milling	DBP	
2	Replica			Up milling	UBP	
				Down milling	DBP	
3	Trial	Sanding	30 s	Up milling	UBS	RaS
				Down milling	DBS	
4	Replica			Up milling	UBS	
				Down milling	DBS	

**Table 2:** Analysis of variance for the burr heights.

	UBP	DBP	UBS	DBS
Factor	*p*-Value			
Sample (A)	0.013	0.000	0.640	0.034
Deburring (B)	0.000	0.000	0.000	0.000
Slot (C)	0.000	0.000	0.000	0.000
AB	0.126	0.577	0.377	0.019
AC	0.010	0.007	0.198	0.008
BC	0.002	0.010	0.000	0.000
ABC	0.001	0.000	0.248	0.010

**Table 3:** Analysis of variance for surface roughness.

	RaP	RaS
Factor	*p*-Value	
Sample (A)	0.073	0.139
Deburring (B)	0.409	0.010
Slot (C)	0.000	0.000
AB	0.000	0.020
AC	0.000	0.004
BC	0.000	0.448
ABC	0.000	0.682

## Data Availability

The data supporting the findings of this study are available from the corresponding author upon reasonable request.

## Abbreviations

The following abbreviations are used in this manuscript:

EDM	Electrical Discharge Machining
In718	Inconel 718
UBP	Up-Milling Burr Height for Polishing Deburring
DBP	Down-Milling Burr Height for Polishing Deburring
UBS	Up-Milling Burr Height for Sanding Deburring
DBS	Down-Milling Burr Height for Sanding Deburring
RaP	Ra Results for Polishing Deburring
RaS	Ra Results for Sanding Deburring

## References

[1] Jain V.K., Jain P.K., Rao P.V. (2012). Editorial: Micromachining. Int. J. Adv. Manuf. Technol..

[2] Takács M., Verö B., Mészáros I. (2003). Micromilling of Metallic Materials. J. Mater. Process. Technol..

[3] Gołaszewski M., Wojciechowski S., Powałka B., Husár J. (2025). Prediction of Surface Topography Anomalies during Radial Immersion Micromilling. Int. J. Mech. Sci..

[4] Gonçalves M.C.C., Alsters R., Curtis D., M’Saoubi R., Ghadbeigi H. (2024). Evolution of Surface Quality in Micromilling Ti-6A1-4V Alloy with Increasing Machined Length. Procedia CIRP.

[5] Mativenga P. (2018). Micromachining. CIRP Encyclopedia of Production Engineering.

[6] Câmara M.A., Rubio J.C.C., Abrão A.M., Davim J.P. (2012). State of the Art on Micromilling of Materials, a Review. J. Mater. Sci. Technol..

[7] Filiz S., Conley C.M., Wasserman M.B., Ozdoganlar O.B. (2007). An Experimental Investigation of Micro-Machinability of Copper 101 Using Tungsten Carbide Micro-Endmills. Int. J. Mach. Tools Manuf..

[8] Sun W., Chen Y.Q., Luo G.A., Zhang M., Zhang H.Y., Wang Y.R., Hu P. (2016). Organs-on-Chips and Its Applications. Chin. J. Anal. Chem..

[9] Aramcharoen A., Mativenga P.T. (2009). Size Effect and Tool Geometry in Micromilling of Tool Steel. Precis. Eng..

[10] Aslantas K., Çiçek A. (2018). The Effects of Cooling/Lubrication Techniques on Cutting Performance in Micro-Milling of Inconel 718 Superalloy. Procedia CIRP.

[11] Fu M.W., Wang J.L. (2021). Size Effects in Multi-Scale Materials Processing and Manufacturing. Int. J. Mach. Tools Manuf..

[12] Chae J., Park S.S., Freiheit T. (2006). Investigation of Micro-Cutting Operations. Int. J. Mach. Tools Manuf..

[13] Brito L.C., Gomes M.C., de Oliveira D., Bacci da Silva M., Viana Duarte M.A. (2023). Vibration Features for Indirect Monitoring of End Micromilling Process. Precis. Eng..

[14] Dib M.H.M., Duduch J.G., Jasinevicius R.G. (2018). Minimum Chip Thickness Determination by Means of Cutting Force Signal in Micro Endmilling. Precis. Eng..

[15] Silva L.C., da Silva M.B. (2019). Investigation of Burr Formation and Tool Wear in Micromilling Operation of Duplex Stainless Steel. Precis. Eng..

[16] Saha S., Ansary S.I., Deb S., Bandyopadhyay P.P. (2023). Influence of Tool Wear on Chip-like Burr Formation during Micro-Milling, and Image Processing Based Measurement of Inwardly-Deflected Burrs. Wear.

[17] Gillespie L.K. (1999). Deburring and Edge Finishing Handbook.

[18] Cao Y., Deng B., Li C., Lu S., Tang Y., Deng S., Wang L., Xie Y. (2025). Burr Formation Mechanisms and Parameter Effects in High-Speed Micro-Milling of Aviation-Grade Soft Metals. J. Manuf. Process..

[19] Ahmed F., Ahmad F., Kumaran S.T., Danish M., Kurniawan R., Ali S. (2023). Development of Cryogenic Assisted Machining Strategy to Reduce the Burr Formation during Micro-Milling of Ductile Material. J. Manuf. Process..

[20] Medeossi F., Sorgato M., Bruschi S., Savio E. (2018). Novel Method for Burrs Quantitative Evaluation in Micro-Milling. Precis. Eng..

[21] Khan K., Varghese A., Dixit P., Joshi S.S. (2019). Effect of Tool Path Complexity on Top Burrs in Micromilling. Procedia Manuf..

[22] Sun S.F., Yin A.C., Wang P.P., Zhang Q.D. (2014). Experimental Study of Micro Milling Burr Control Based on Process Parameters Optimization. Appl. Mech. Mater..

[23] Mori M., Nagasuna T., Hamada H. (2016). The Difference in Micro-Deburring Finish Produced by Groove Cutting Method. Lecture Notes in Computer Science (Including Subseries Lecture Notes in Artificial Intelligence and Lecture Notes in Bioinformatics).

[24] Sun Q., Cheng X., Zhao G., Yang X., Zheng G. (2019). Experimental Study of Micromilling Burrs of 304 Stainless Steel. Int. J. Adv. Manuf. Technol..

[25] Matsumura T., Konno T., Tobe S., Komatsu T. (2011). Deburring of Micro-Scale Structures Machined in Milling. ASME 2010 International Manufacturing Science and Engineering Conference, MSEC 2010.

[26] Kienzler A., Deuchert M., Schulze V. (2010). Burr Minimization and Removal by Micro Milling Strategies or Micro Peening Processes. Burrs—Analysis, Control and Removal.

[27] Kumar A.S., Deb S., Paul S. (2022). Burr Removal from High-Aspect-Ratio Micro-Pillars Using Ultrasonic-Assisted Abrasive Micro-Deburring. J. Micromech. Microeng..

[28] Jeong Y.H., HanYoo B., Lee H.U., Min B.K., Cho D.W., Lee S.J. (2009). Deburring Microfeatures Using Micro-EDM. J. Mater. Process. Technol..

[29] Liu Y., Xu D., Agmell M., Saoubi R.M., Ahadi A., Stahl J.E., Zhou J. (2021). Numerical and Experimental Investigation of Tool Geometry Effect on Residual Stresses in Orthogonal Machining of Inconel 718. Simul. Model. Pract. Theory.

[30] Ucun I., Aslantas K., Bedir F. (2013). An Experimental Investigation of the Effect of Coating Material on Tool Wear in Micro Milling of Inconel 718 Super Alloy. Wear.

[31] Fang B., Yuan Z., Li D., Gao L. (2021). Effect of Ultrasonic Vibration on Finished Quality in Ultrasonic Vibration Assisted Micromilling of Inconel718. Chin. J. Aeronaut..

[32] de Paiva Silva G., Bacci da Silva M., de Oliveira D. (2023). Influence of Abrasive Deburring in Indirect Tool Wear Measurement in Micromilling of Inconel 718. J. Braz. Soc. Mech. Sci. Eng..

[33] ASM (1993). ASM Handbook Volume 2—Properties and Selection: Nonferrous Alloys and Special-Purpose Materials. ASM Metals Handbook.

[34] Wang F., Cheng X., Liu Y., Yang X., Meng F. (2017). Micromilling Simulation for the Hard-to-Cut Material. Procedia Eng..

[35] Reed R.C. (2006). The Superalloys: Fundamentals and Applications. The Superalloys: Fundamentals and Applications.

[36] (1996). Geometrical Product Specifications (GPS)—Surface Texture: Profile Method—Rules and Procedures for the Assessment of Surface Texture 1996, 8.

[37] (2002). Especificações Geométricas Do Produto (GPS)—Rugosidade: Metódo Do Perfil—Termos, Definições e Parâmetros Da Rugosidade. Étricas Do Produto (GPS)—Rugosidade: Metódo Do Perfil—Termo.

[38] Liang J.L., Zhang F., Zhang J.H., Huang W.Q., Wen Y.X., Chen B.C. (2023). Improvement of Mesh Atomizer Performance by Electrolytic Polishing. Appl. Sci..

[39] Zhang Z., Wang C., Guo J., Cheung C.F. (2023). Micro-Milling Tool Mark Removal Mechanism by Fluid Jet Polishing and Its Application for the Precision Manufacturing of Micro-Fluidic Chip Molds. Tribol. Int..

[40] Hong X., Jiang C., Ye H., Jiang Z., Liu J., Sun L. (2025). Effects of Magnetic Compound Fluid Polishing on Surface Morphology and Laser Damage Resistance of Multilayer Dielectric Pulse Compression Gratings. Opt. Laser Technol..

[41] Marinescu I.D., Rowe W.B., Dimitrov B., Inasaki I. (2004). Tribology of Abrasive Machining Processes.

[42] Hutchings I., Shipway P. (2017). Tribology: Friction and Wear of Engineering Materials.

[43] Qu D., Xu Z., Song Y., Ma X., Ji J. (2025). Burrs at Exit Position in Slot Micro-Milling Considering Tool Runout: Feature Extraction, Characterization, and Modeling. J. Manuf. Process..

[44] Vipindas K., Kuriachen B., Mathew J. (2019). Investigations into the Effect of Process Parameters on Surface Roughness and Burr Formation during Micro End Milling of TI-6AL-4V. Int. J. Adv. Manuf. Technol..

[45] Ziberov M., de Oliveira D., da Silva M.B., Hung W.N.P. (2020). Wear of TiAlN and DLC Coated Microtools in Micromilling of Ti-6Al-4V Alloy. J. Manuf. Process..

[46] Wu M., Wu B., Zhang S., Parmar A., Liu W., Shin Y.C. (2025). Laser-Induced Plasma Micro Deburring: Time-Resolved Observation and Workpiece Characterization. J. Manuf. Process..

[47] Ucun I., Aslantas K., Bedir F. (2015). The Performance Of DLC-Coated and Uncoated Ultra-Fine Carbide Tools in Micromilling of Inconel 718. Precis. Eng..

[48] Chern G.L., Wu Y.J.E., Cheng J.C., Yao J.C. (2007). Study on Burr Formation in Micro-Machining Using Micro-Tools Fabricated by Micro-EDM. Precis. Eng..

[49] de Oliveira Campos F., Araujo A.C., Jardini Munhoz A.L., Kapoor S.G. (2020). The Influence of Additive Manufacturing on the Micromilling Machinability of Ti6Al4V: A Comparison of SLM and Commercial Workpieces. J. Manuf. Process..

